# Exploring the biocatalytic potential of wild-type split transketolases

**DOI:** 10.1039/d6cb00243a

**Published:** 2026-10-07

**Authors:** Alessia Tonoli, Silvia Anselmi, Yeke Ni, John M. Ward, Helen C. Hailes, Jack W. E. Jeffries

**Affiliations:** a Department of Biochemical Engineering, University College London Bernard Katz Building, Gower Street London WC1E 6BT UK jack.jeffries.12@ucl.ac.uk; b Department of Chemistry, University College London 20 Gordon Street London WC1H 0AJ UK h.c.hailes@ucl.ac.uk; c Manufacturing Futures Laboratory, University College London 7 Sidings Street London E20 2AE UK

## Abstract

Split transketolases (split TKs) are an underexplored class of carboligases with potential for application in biocatalysis. Here, we investigate ten split TKs and reveal substantial functional heterogeneity, with marked differences in substrate scope, activity, enantioselectivity, and robustness. The enzymes accepted diverse acceptors, aliphatic and aromatic, while retaining the canonical (*S*)-selectivity of full-length TKs. Pyruvate acceptance further revealed distinct catalytic behaviours, including cross-coupling and self-coupling specificity. Selected enzymes also displayed high organic solvent tolerance. Notably, pQR2829 combined high activity, strong stereocontrol, and cross-coupling specificity, whereas pQR2809 showed exceptional robustness and broad substrate acceptance. Overall, these findings establish split TKs as a diverse and promising class of carboligases for further biocatalytic development and engineering.

## Abstract 2

A panel of split transketolases reveals diverse catalytic behaviours, combining distinct cross- and self-coupling specificities with conserved (*S*)-selectivity and differences in activity, substrate scope, and robustness.

## Graphical abstract

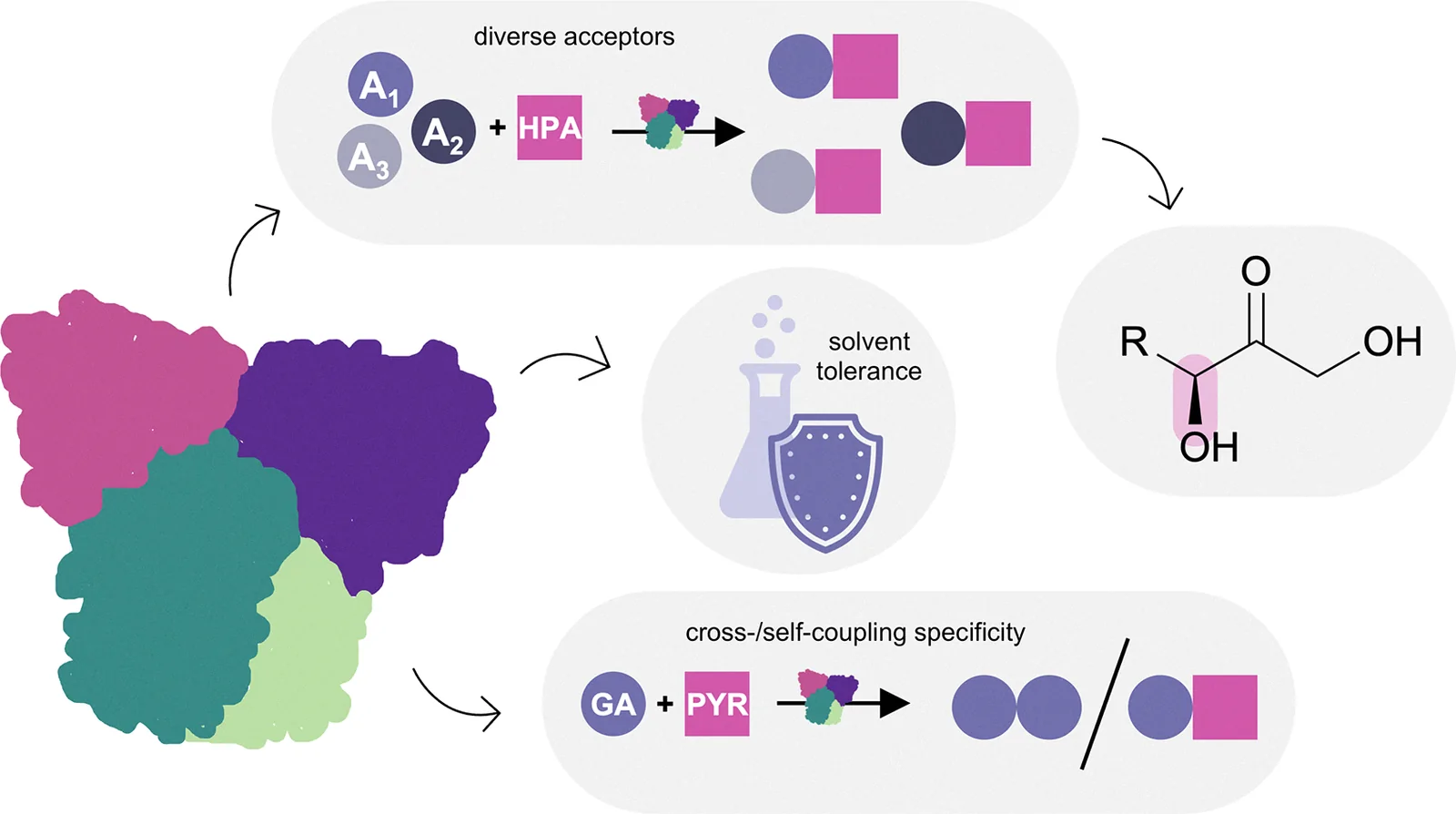

## Introduction

Transketolases (TKs) are thiamine diphosphate (ThDP)-dependent enzymes that catalyse the stereoselective transfer of a two-carbon ketol unit from a donor substrate to a carbonyl acceptor.^1^ Beyond their central physiological roles in the pentose phosphate pathway and the Calvin cycle, TKs are attractive biocatalysts due to their ability to stereoselectively form carbon–carbon bonds under mild reaction conditions. Consequently, several TKs, including the one from *Escherichia coli* (*Eco*TK), as well as the thermostable TK from *Geobacillus stearothermophilus* (*Gst*TK), have been extensively studied and engineered for a variety of applications, including the improved acceptance of non-natural substrates,^2–9^ and the enhancement or reversal of stereoselectivity.^2,10,11^ The use of β-hydroxypyruvate (HPA, 1) as a donor is particularly attractive because it is readily accepted by many TKs and its decarboxylation drives product formation.^12^

Most characterised TKs are homodimeric enzymes in which the active site is formed at the interface between two identical subunits. However, an alternative architecture exists in which the canonical TK domains are encoded on two separate polypeptides. These so-called split TKs include the enzyme from the hyperthermophilic bacterium *Carboxydothermus hydrogenoformans* (*Chy*TK), for which a crystal structure has been determined, revealing assembly into a functional heterotetramer.^13^*Chy*TK displays remarkable tolerance to elevated temperatures and high concentrations of organic co-solvents, retaining full activity after 1 hour of incubation at temperatures up to 70 °C, as well as 40–60% of its activity after 1 hour in 50% (v/v) organic co-solvents. These properties underscore its potential for biocatalytic applications.

In our previous work, we expanded the known sequence space of split TKs through genome and metagenome mining, identifying new representatives and showing that this class is considerably more widespread and diverse than previously recognised. Ten enzymes were successfully produced and characterised, revealing substantial variation in stability and activity towards HPA 1 and glycolaldehyde (GA, 2a). The study also identified additional thermostable split TKs beyond *Chy*TK.^14^

In contrast to full-length TKs, whose substrate scope and engineering potential have been extensively investigated, relatively little is still known about the acceptance of non-native substrates by split TKs and systematic comparisons across multiple split TKs remain scarce. For *Chy*TK, activity has been reported with phenylacetaldehyde and cyclohexanecarboxaldehyde as acceptors. However, product formation was not confirmed, and enzyme performance was not quantified.^13^

Here, we investigate the substrate scope and stereoselectivity of ten wild-type split TKs (Table S1) using a panel of aliphatic and aromatic aldehyde acceptors as well as the alternative donor pyruvate (PYR, 4). By comparing enzymes originating from diverse phylogenetic and environmental backgrounds, we identify marked differences in substrate acceptance, cross-coupling specificity, and stereoselectivity. The results highlight a subset of split TKs as promising candidates for future biocatalytic applications.

## Results and discussion

### Acceptor scope and stereoselectivity of split TKs

The activity of split TKs was first evaluated using HPA 1 as ketol donor and the non-hydroxylated aliphatic aldehydes propanal (2b) and pentanal (2c) as acceptors (Scheme 1). Full-length TKs can accept both aldehydes, although typically with lower efficiency than hydroxylated acceptors such as GA 2a.^11,15^ Reactions were performed with purified enzymes (0.5 mg mL^−1^) for 20 h at 30 °C, as a compromise to allow comparison between enzymes with different temperature preferences and stabilities, while considering the limited stability of 1.^16^ Product formation was quantified by chiral GC-FID, following peracetylation of the corresponding ketodiols. Racemic standards were prepared using the TK biomimetic reaction^2,4,17^ and used for calibration purposes.

**Scheme 1:**
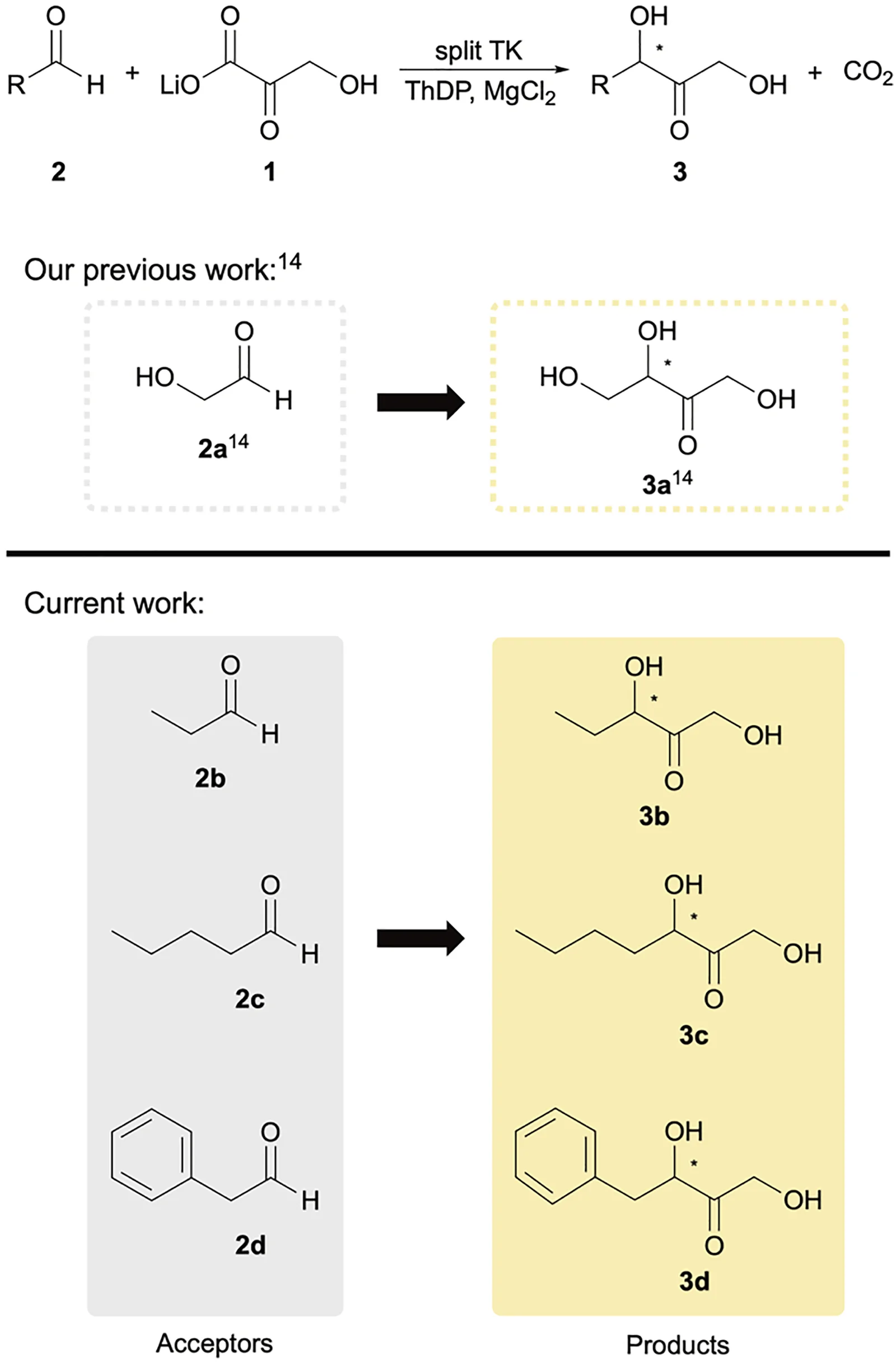
Split TK-catalysed reactions with HPA (1) as ketol donor, and different aldehyde acceptors (2). Split TKs were tested in combination with 1 and various acceptors: GA (2a) in previous work,^14^ while propanal (2b), pentanal (2c), and phenylacetaldehyde (2d) in this work.

When tested with 20 mM acceptor, all split TKs displayed limited activity toward the shorter aldehyde 2b, with product yields below 6% (Fig. 1). In contrast, most enzymes showed substantially improved activity with the longer chain aldehyde 2c. Among the enzymes tested, pQR2829, derived from the metagenome of a salt mine, was the most active when using 20 mM acceptor, yielding 5% GC yield with 2b and 39% with 2c. Other active split TKs, namely pQR2807, pQR2809, pQR2823 and pQR2830, produced only low amounts of product 3b (1–4%) but achieved higher conversions for 3c (8–31%). In contrast, pQR2812, which displayed activity in the reaction with 1 and 2a,^14^ showed little or no activity toward either substrate, while pQR2825, pQR2827 and pQR2828 were inactive under the condition tested. Interestingly, the chimera pQR2807 and its wild-type version pQR2834 exhibited comparable activity with 2b, whereas pQR2807 generated approximately a five-fold higher yield with 2c by GC-analysis.

**Fig. 1:**
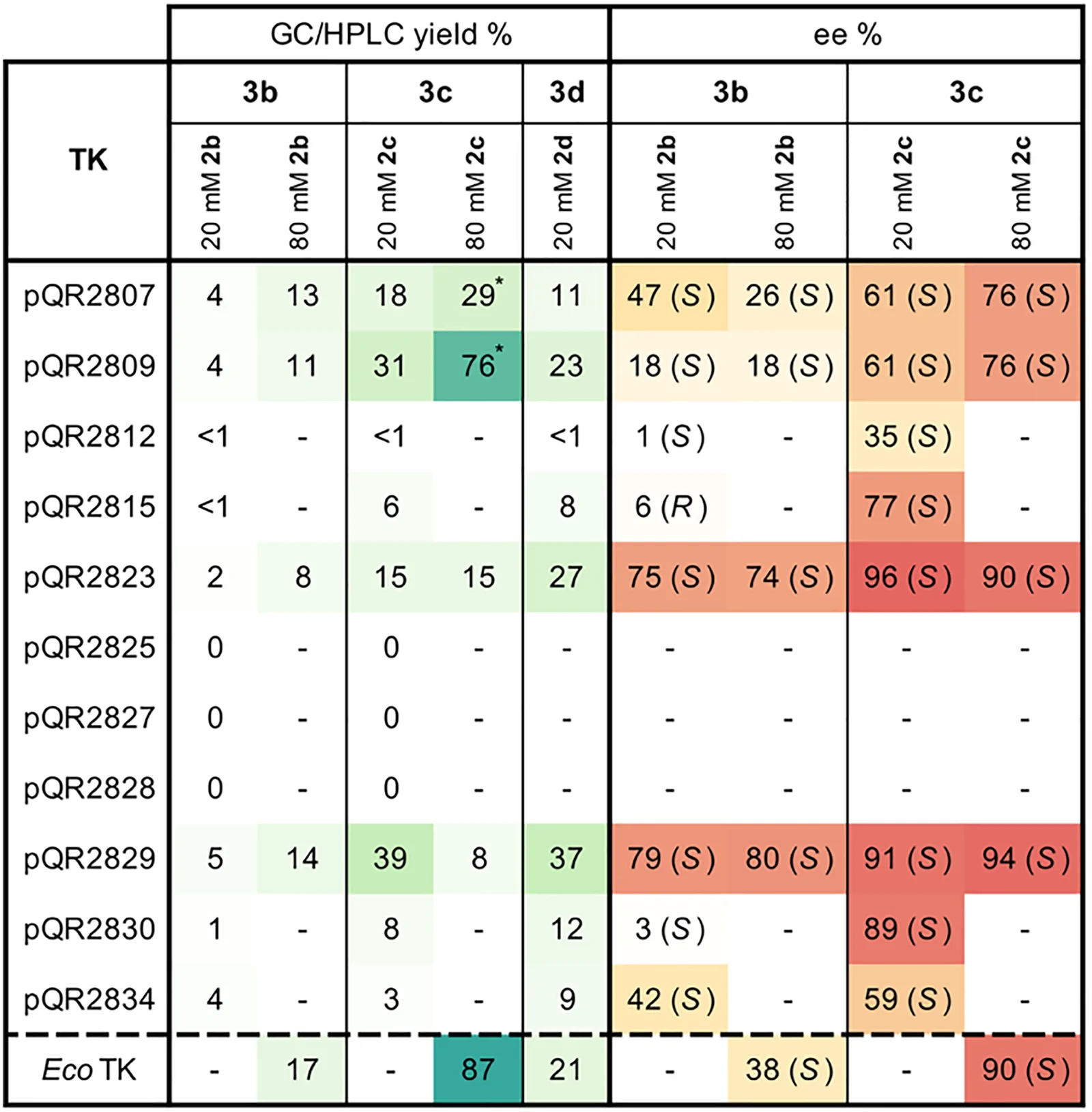
Summary of analytical yields and enantiomeric excess (ee) of the investigated split TK-catalysed reactions with HPA 1 and aldehyde acceptors 2b–d. Reactions contained 32 mM 1, 20–80 mM 2b–d, 2.4 mM ThDP, 9 mM MgCl_2_, and 0.5 mg mL^−1^ of purified split TK in 50 mM Tris–HCl pH 7.2. Full-length *Eco*TK was included as reference. Reactions were performed at 30 °C for 20 hours in triplicate (or in duplicate for reactions containing 80 mM acceptor). For GC, reactions were peracetylated prior to analysis. HPLC yields were calculated based on calibration curves of the corresponding products, while GC yields were calculated based on calibration curves of the corresponding peracetylated products. Standard deviations (SD) were <3%, unless otherwise indicated by an asterisk (*), for which SD were <10%.

The consistently higher yields obtained with 2c suggested that some split TKs may preferentially accept longer-chain aliphatic aldehydes. However, because 2b is considerably more volatile than 2c, reduced conversions could also reflect some substrate loss during the reaction. To further investigate the acceptance of these substrates, selected enzymes were re-evaluated using an excess of aldehyde (80 mM) while maintaining the donor concentration at 32 mM, to assess whether higher yields could be obtained; full-length *Eco*TK was included as a benchmark TK. Under these conditions, pQR2807 and pQR2809 displayed improved conversions with both substrates and continued to perform better with 2c than with 2b (Fig. 1). In contrast, pQR2829 and pQR2823 showed increased yields with 2b but not with 2c, suggesting that elevated aldehyde concentrations may have detrimental effects on activity or stability. Nevertheless, pQR2829 remained the most active split TK with 2b, whereas pQR2809 achieved the highest conversion with 2c, reaching 76% GC yield. *Eco*TK outperformed all split variants, yielding 17% and 86% yields by GC-analysis with 2b and 2c, respectively. Although the present data do not conclusively establish whether the observed preference for 2c reflects intrinsic substrate selectivity, they demonstrate that several split TKs retain substantial activity toward non-hydroxylated aliphatic aldehydes.

Similar to split TKs, full-length TKs from *Geobacillus stearothermophilus* (*Gst*TK) and *Thermotoga maritima* (*Tma*TK) have been reported to exhibit higher activity toward C_5_ than C_3_ aldehyde acceptors.^18,19^ Variable results have instead been reported for *Eco*TK, with higher yields reported for either 2b or 2c acceptors depending on the study.^2,10,20,21^ Under the conditions used in our experiments, *Eco*TK showed higher activity toward the C_5_ acceptor.

GC analysis for yield determination, performed using a chiral column, also enabled determination of the stereoselectivity of the split TK-catalysed reactions (Fig. 1). In almost all cases, the major product corresponded to the (*S*)-enantiomer, similarly to full-length TKs.^10,16^ Across the enzyme set, higher stereoselectivities were generally observed with 2c than with 2b. Among the split TKs tested, pQR2823 and pQR2829 displayed the highest stereoselectivities. With 2b, these enzymes yielded enantiomeric excesses (ees) of up to 75% and 80%, respectively, increasing to 96% and 94% with 2c as the acceptor.

High selectivity toward 2c was also observed for pQR2830 (89% ee), while exhibiting almost no stereoselectivity with 2b (3% ee). Other split TKs, including pQR2807, pQR2809, pQR2815 and pQR2834, exhibited moderate selectivity with 2c, ranging from 59% to 77% ee.

Similarly to split TKs, *Eco*TK has been reported to yield higher stereoselectivity with the C_5_ acceptor 2c over shorter substrates, despite variability in yields.^2,10,20^ Notably, pQR2823 and pQR2829 exhibit stereoselectivities toward 2b that exceed those reported for *Eco*TK. In particular, pQR2829 reached 80% ee, whereas *Eco*TK has been reported to achieve up to 58% ee^20^ (38% ee in our experiment). With 2c, the stereoselectivities of these split TKs were comparable to, or slightly higher than, those reported for *Eco*TK. Despite this, lower overall yields were obtained with the split TKs compared to *Eco*TK for both substrates. Conversely, pQR2809, which achieved the highest conversion with 2c among the split TKs and approached the performance of *Eco*TK, displayed lower stereoselectivity (up to 76% ee). Unlike other split TKs and full-length TKs, pQR2809 contains a leucine residue at the position equivalent to the *Eco*TK Asp469, a well-established hotspot linked to substrate specificity and stereoselectivity in full-length TKs.^4,5,9,22^

Relative to *Gst*TK, pQR2823 and pQR2829 show comparable stereoselectivities.^11,15^ However, since *Gst*TK data were obtained using trifluoroacetylation while this study used peracetylation (which has been reported to induce a partial racemisation), the stereoselectivity of the split TKs may be underestimated (5% reduction in measured ee reported for the derivatisation of 3b using acetylation *versus* trifluoroacetylation).^15^

Overall, the data demonstrate that the split TK architecture preserves the stereochemical fidelity characteristic of full-length TKs while, in some cases, enabling higher levels of stereoselectivity. Together with the activity data, these results indicate that the enzymes generally perform poorly with the short-chain acceptor 2b, whereas both longer aliphatic acceptor 2c or the hydroxylated acceptor 2a^14^ are preferred.

The substrate scope of the split TKs was further explored using the aromatic acceptor phenylacetaldehyde (2d) in combination with HPA 1 (Scheme 1). The split *Chy*TK has been reported to accept 2d, but without confirmed product identification or activity quantification.^13^ Wild-type full-length TKs also accept certain aromatic substrates with HPA 1 as donor, though activity is substantially lower than with hydroxylated substrates.^4,6^ Reactions were performed under the same conditions employed for the aliphatic substrates, with the addition of 10% (v/v) DMSO to improve substrate solubility, and analysed by HPLC. Quantification using a product standard showed that pQR2829 was the most active enzyme, yielding 37% yield by HPLC analysis, followed by pQR2823 (27%) and pQR2809 (23%) (Fig. 1). Under the conditions employed, all three enzymes outperformed *Eco*TK (21%). The native sequence of pQR2809 includes amino acids equivalent to the *Eco*TK variants L382A and D469L, both associated with aromatic substrate acceptance in full-length TKs.^5,6,23^ Conversely, pQR2829 does not contain any of these substitutions. The presence of additional peaks in all samples suggests side-product formation. Although previously observed for other aromatic acceptors with donor 1,^4^ no by-products were reported for 2d.

To further characterise the most active enzyme, the progress of pQR2829-catalysed reaction was monitored, yielding a specific activity of 0.058 ± 0.001 µmol min^−1^ mg^−1^.

### Pyruvate acceptance and catalytic specificity of split TKs

To probe donor promiscuity in the split TKs, their ability to utilise PYR (4) as an alternative donor was evaluated using GA (2a) as acceptor (Scheme 2). PYR utilisation is characteristic of another ThDP-dependent enzyme of the TK-like family, named 1-deoxy-d-xylulose-5-phosphate synthase (DXS), able to catalyse analogous carbon–carbon bond formation reactions using 4 and d-glyceraldehyde-3-phosphate as substrates.^24^ While TK and DXS share the same domain order in their primary structures, their active-site architectures differ: in DXSs the active site is composed of amino acids from a single polypeptide chain, while in full-length TK homodimers it is formed by residues from both subunits. Regarding the donor-binding pocket, the main difference is a conserved histidine in TKs (*Eco*TK H100), which is most commonly replaced by a phenylalanine or a tyrosine in DXS enzymes.^7^

**Scheme 2:**
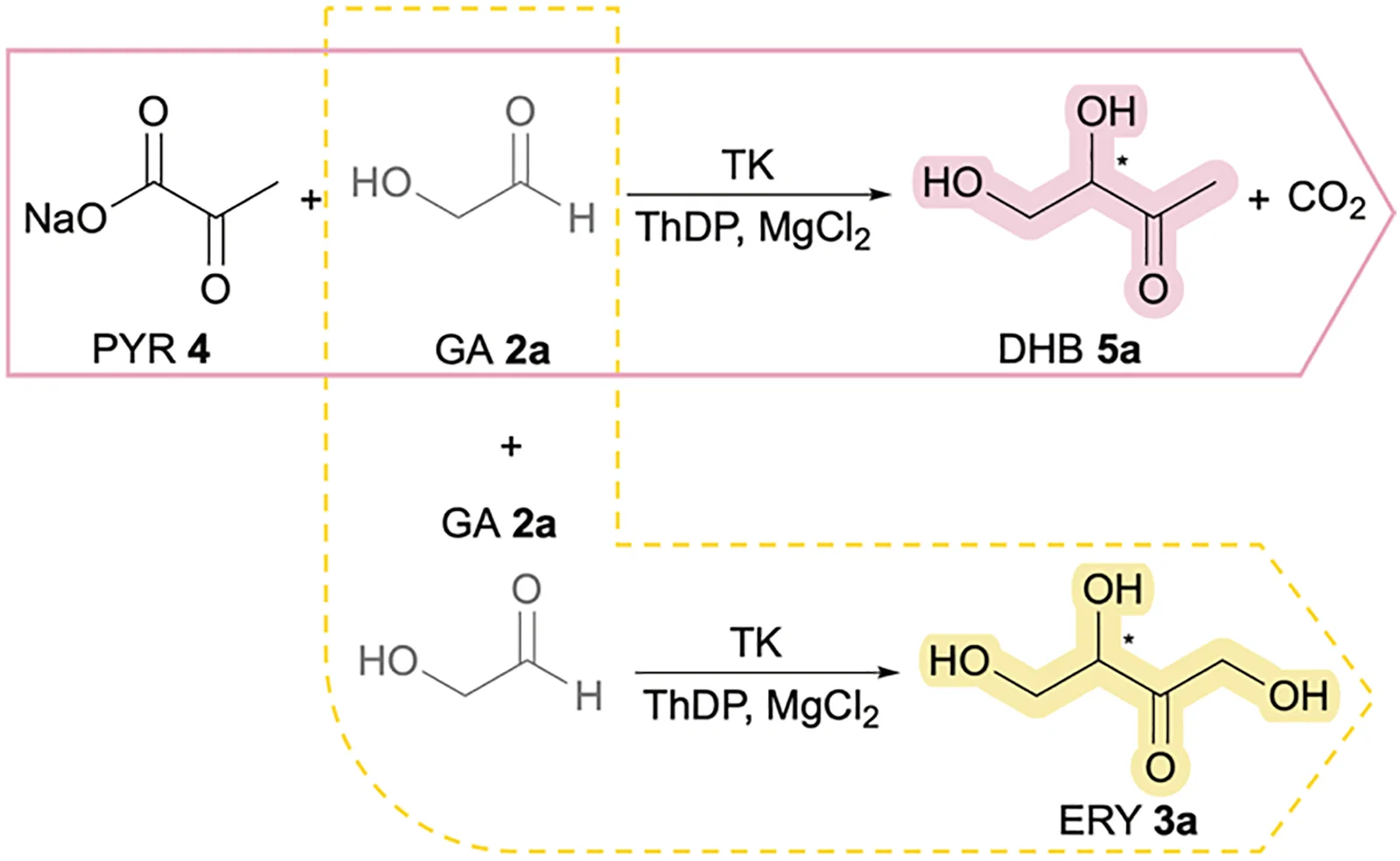
TK-catalysed reaction with (PYR, 4) as donor and GA 2a as acceptor. The desired cross-acyloin condensation (pink arrow) product is DHB (5a). However, acceptor self-coupling can occur (yellow arrow), producing the side-product erythrulose (ERY, 3a).

All split TKs investigated in this study retain the canonical histidine at the corresponding position.

Previous studies have shown that full-length TKs can accept 4 as donor, but with substantially lower yields than when the hydroxylated donor 1 is used.^7^ Using HPLC to analyse the *Eco*TK-catalysed reaction with 4 and 2a, Yu and co-workers observed that formation of the cross-coupling product 3,4-dihydroxybutan-2-one (DHB, 5a) was accompanied by the side product erythrulose (ERY, 3a) arising from a competing self-coupling reaction^9^ (Scheme 2). They further noted that it remains unclear whether this by-product was formed by other TKs, as the assays used in previous studies monitored only the decarboxylation step of the reaction.

In the present study, the self-coupling reaction was also observed for the full-length TK from *Thermotoga maritima* (*Tma*TK) (Fig. 3). In contrast, split ChyTK has been reported not to accept 4 as donor.^13^

**Fig. 2:**
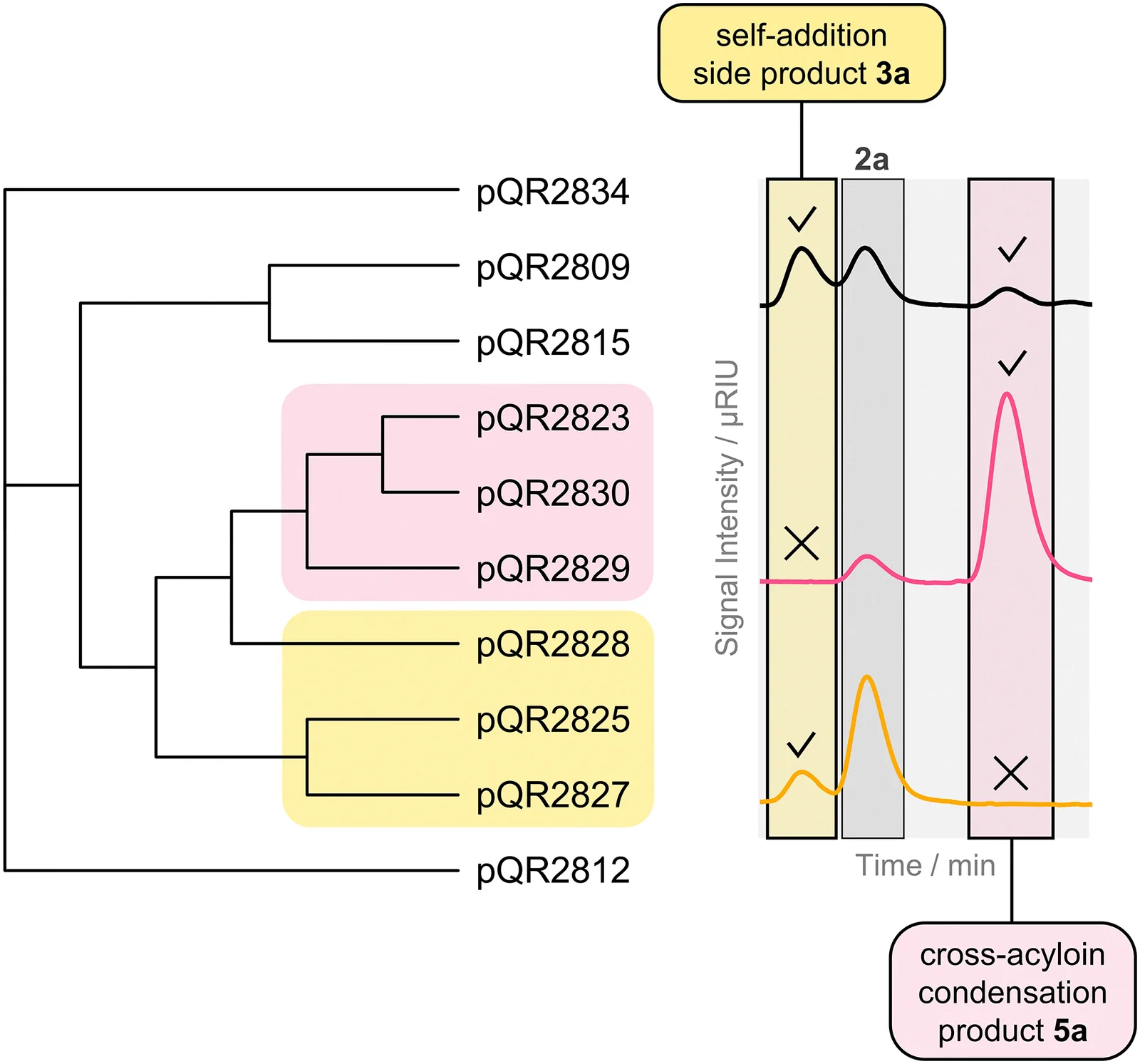
Cladogram illustrating the phylogenetic clustering of characterised split TKs in relation to their cross-/self-coupling specificity in the reaction with PYR 4 and GA 2a, together with corresponding chromatograms showing the three observed outcomes: formation of the desired cross-coupling product DHB 5a only (pink); formation of the self-coupling product ERY 3a only (yellow); formation of both products (black).

**Fig. 3:**
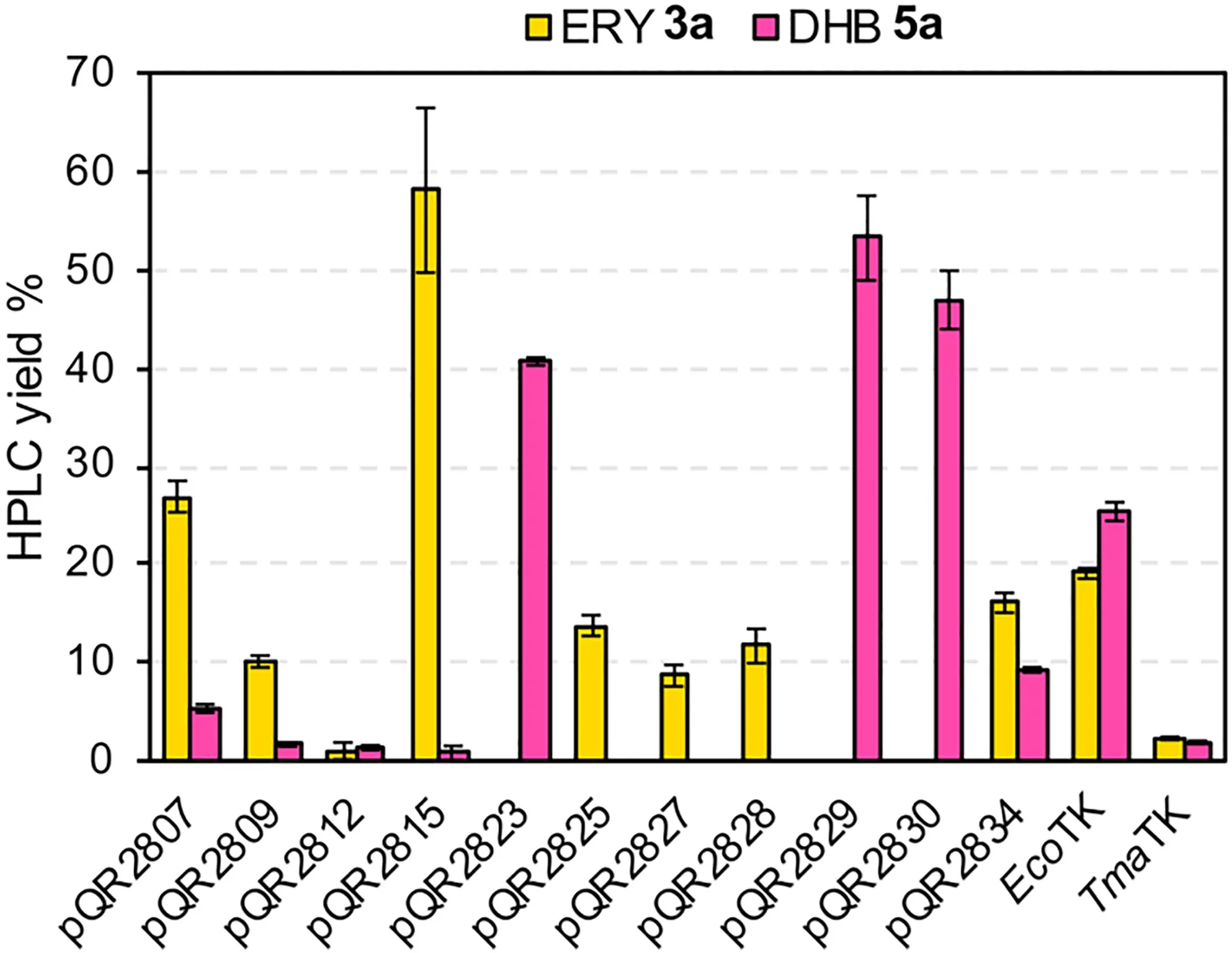
HPLC yields of split TK-catalysed reactions with PYR 4 and GA 2a. The reactions were run in triplicate for 20 hours at 30 °C using 0.5 mg mL^−1^ of purified split TKs. The reactions contained 40 mM 4, 20 mM 2a, 2.4 mM ThDP, 9 mM MgCl_2_, in 50 mM Tris–HCl pH 7.2. Full-length *Eco*TK (0.5 mg mL^−1^) and *Tma*TK (0.2 mg mL^−1^) were also included as reference.

Here, reactions with 4 and 2a catalysed by split TKs were analysed by HPLC-RI following incubation for 20 h at 30 °C. Three distinct catalytic behaviours were observed (Fig. 3). A first group of enzymes (pQR2807, pQR2809, pQR2812, pQR2815 and pQR2834) catalysed formation of both the desired cross-acyloin product 5a and the self-coupling product 3a. A second group (pQR2823, pQR2829 and pQR2830) produced exclusively 5a without detectable by-products, indicating cross-coupling specificity. Interestingly, while His473Asn *Eco*TK is the only reported variant to yield 5a without by-products,^9^ the split TKs in this cross-coupling-specific subgroup retain the canonical histidine at the corresponding position. A third group of split TKs (pQR2825, pQR2827 and pQR2828) formed 3a with no detectable cross-coupling activity. These enzymes correspond to metagenome-derived split TKs that showed only trace or no activity in all other reactions previously tested,^14^ suggesting a distinct substrate specificity/preference compared to the other characterised TKs, both full-length and split.

Notably, phylogenetic analysis revealed a clear correlation between sequence clustering and catalytic behaviour: enzymes in the cross-specific group cluster together, while the self-coupling-only enzymes are found in neighbouring branches in the cladogram (Fig. 2).

### Organic solvent tolerance of split TKs

Tolerance to process-related conditions, including high concentration of organic solvents, is a desirable property for industrial biocatalysts. Given their previously observed operational and thermal stability,^14^ the bacterial split TKs pQR2807 and pQR2809 were further evaluated for activity in the presence of increasing concentrations of DMSO, a co-solvent frequently used for substrate solubilisation. *Eco*TK was included as reference. Enzymes activity was assessed after a 6 h reaction with 1 and 2d, and product yields determined by HPLC were expressed relative to the benchmark condition containing 10% (v/v) DMSO, as it was used in previous assays with the same substrates. All three enzymes showed a progressive loss of activity (Fig. 4). pQR2807, a chimera of *Fusobacterium nucleatum* subspecies *nucleatum* and *polymorphum*, was outperformed by *Eco*TK (68% *versus* 74% remaining activity at 20% (v/v) DMSO, and 30% *versus* 43% at 40% (v/v) DMSO). pQR2809 was the most solvent-tolerant, retaining 94% activity at 20% (v/v), and 64% at 40% (v/v) DMSO, demonstrating good tolerance to high organic solvent content. This stability is particularly notable in combination with its previously observed thermostability^14^ and broad substrate scope, highlighting pQR2809 as a strong candidate for further biocatalytic development. *Chy*TK was previously reported to retain approximately 60% residual activity following a 1 h exposure to 50% (v/v) DMSO and other organic solvents, although direct comparison is limited by differences in assay conditions and experimental protocol.^13^

**Fig. 4:**
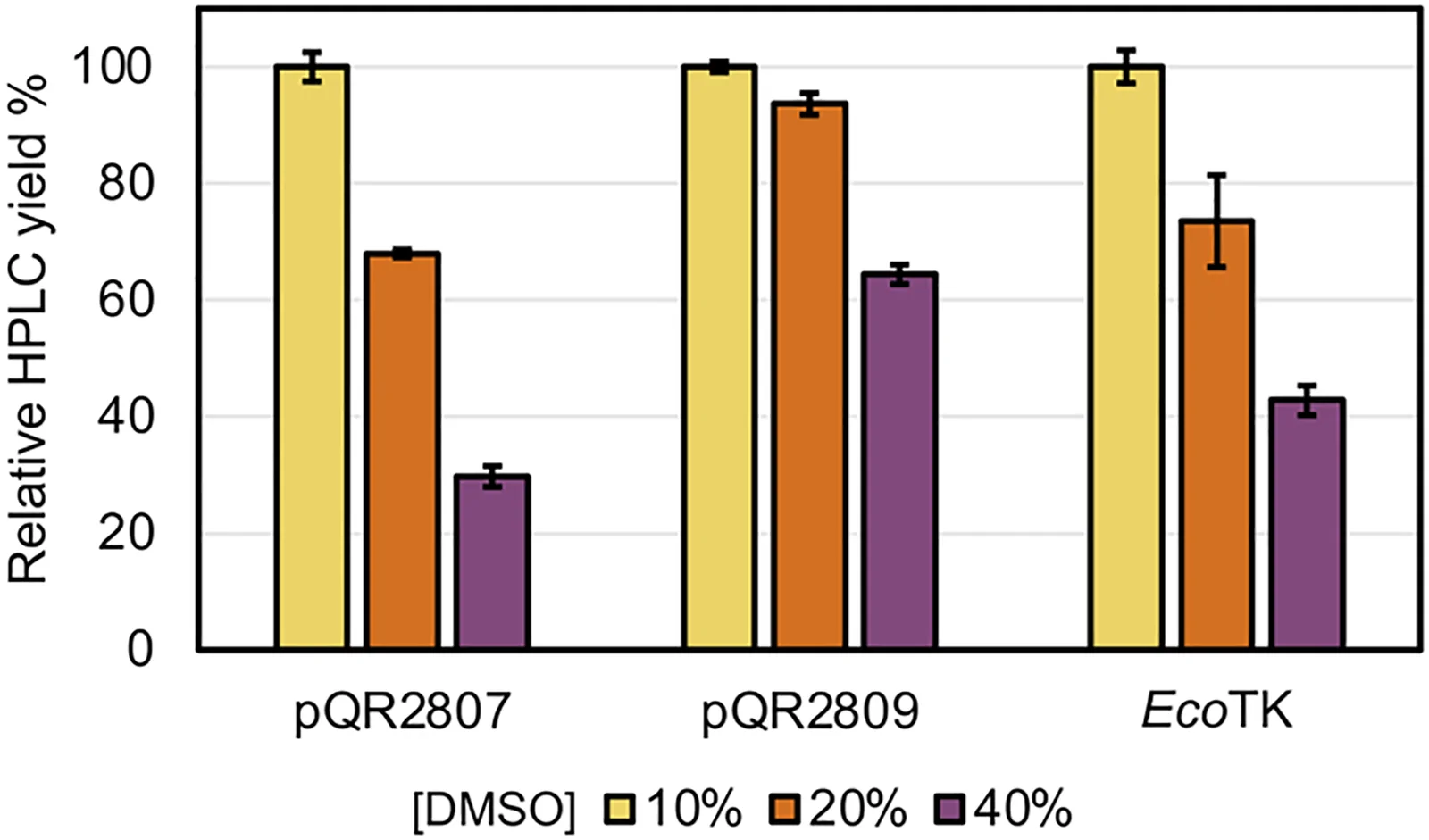
Effect of DMSO concentration on the performance of split TKs, in comparison to *Eco*TK. Relative HPLC yields (%) were based on the product formed following a 6 hour reaction at 30 °C with HPA 1 and 2d, performed in duplicate. Yields were normalised to the reaction containing 10% (v/v) DMSO.

## Conclusions

This study establishes split TKs as a diverse and functionally versatile class of carboligases with significant potential for biocatalysis. While almost all enzymes yield products with the canonical (*S*)-configuration, as observed for full-length TKs, they exhibit enzyme-dependent variation in substrate scope, activity, and selectivity rather than uniform behaviour dictated by their split architecture. While hydroxylated substrates are generally preferred, a subset of enzymes also accepts non-natural aliphatic and aromatic aldehydes, with variable efficiency.

Within the enzyme panel, a subgroup of metagenome-derived split TKs emerges as particularly interesting, combining cross-coupling specificity with enhanced enantioselectivity that in some cases exceeds that of *Eco*TK, underscoring their potential as underexplored biocatalysts. Among these, pQR2829 stands out by integrating higher activities, strong stereocontrol, and cross-coupling specificity, although its performance is limited by reduced stability and possible substrate inhibition. In contrast, pQR2809 displays exceptional robustness and broad substrate tolerance but lower selectivity.

Together, these findings position split TKs as a promising carboligases class. Targeted engineering of high-performing yet limited enzymes, particularly pQR2829 and pQR2809, offers a route to improving activity–stability-selectivity trade-offs and unlocking their synthetic potential.

## Author contributions

Conceptualisation: A. T., J. W. E. J., H. C. H.; methodology: A. T., S. A., Y. N.; validation: A. T., S. A., Y. N.; investigation: A. T., S. A., Y. N.; formal analysis: A. T., S. A., Y. N.; data curation: A. T.; writing – original draft: A. T.; writing – review & editing: A. T., Y. N., J. W. E. J., H. C. H.; supervision: J. W. E. J., H. C. H., J. M. W.; funding acquisition: J. W. E. J., J. M. W., H. C. H.; resources: J. W. E. J., H. C. H., J. M. W.

## Conflicts of interest

There are no conflicts to declare.

## Supplementary Material

CB-OLF-D6CB00243A-s001

## Data Availability

The materials and methods and the data supporting this article have been included as part of the supplementary information (SI) or previously published.^14^ Supplementary information is available. See DOI: https://doi.org/10.1039/d6cb00243a.

## References

[cit1] Schenk G., Duggleby R. G., Nixon P. F. (1998). Int. J. Biochem. Cell Biol..

[cit2] Cazares A., Galman J. L., Crago L. G., Smith M. E., Strafford J., Rios-Solis L., Lye G. J., Dalby P. A., Hailes H. C. (2010). Org. Biomol. Chem..

[cit3] Hibbert E. G., Senussi T., Smith M. E., Costelloe S. J., Ward J. M., Hailes H. C., Dalby P. A. (2008). J. Biotechnol..

[cit4] Galman J. L., Steadman D., Bacon S., Morris P., Smith M. E. B., Ward J. M., Dalby P. A., Hailes H. C. (2010). Chem. Commun..

[cit5] Payongsri P., Steadman D., Strafford J., MacMurray A., Hailes H. C., Dalby P. A. (2012). Org. Biomol. Chem..

[cit6] Saravanan T., Reif M.-L., Yi D., Lorillière M., Charmantray F., Hecquet L., Fessner W.-D. (2017). Green Chem..

[cit7] Saravanan T., Junker S., Kickstein M., Hein S., Link M. K., Ranglack J., Witt S., Lorillière M., Hecquet L., Fessner W. D. (2017). Angew. Chem., Int. Ed..

[cit8] Mukhopadhyay A., Karu K., Dalby P. A. (2024). Sci. Rep..

[cit9] Yu H., Hernández López R. I., Steadman D., Méndez-Sánchez D., Higson S., Cázares-Körner A., Sheppard T. D., Ward J. M., Hailes H. C., Dalby P. A. (2020). FEBS J..

[cit10] Smith M. E. B., Hibbert E. G., Jones A. B., Dalby P. A., Hailes H. C. (2008). Adv. Synth. Catal..

[cit11] Zhou C., Saravanan T., Lorillière M., Wei D., Charmantray F., Hecquet L., Fessner W. D., Yi D. (2017). ChemBioChem.

[cit12] Marsden S. R., Gjonaj L., Eustace S. J., Hanefeld U. (2017). ChemCatChem.

[cit13] James P., Isupov M. N., De Rose S. A., Sayer C., Cole I. S., Littlechild J. A. (2020). Front. Microbiol..

[cit14] Tonoli A., Anselmi S., Ward J. M., Hailes H. C., Jeffries J. W. E. (2025). ChemBioChem.

[cit15] Yi D., Saravanan T., Devamani T., Charmantray F., Hecquet L., Fessner W. D. (2015). Chem. Commun..

[cit16] Abdoul-Zabar J., Sorel I., Hélaine V., Charmantray F., Devamani T., Yi D., De Berardinis V., Louis D., Marlière P., Fessner W. D. (2013). Adv. Synth. Catal..

[cit17] Smith M. E. B., Smithies K., Senussi T., Dalby P. A., Hailes H. C. (2006). Eur. J. Org. Chem..

[cit18] Casajus H., Lagarde A., Leremboure M., Miguel T. D. D., Nauton L., Thery V., Fessner W. D., Duguet N., Charmantray F., Hecquet L. (2020). ChemCatChem.

[cit19] Cárdenas-Fernández M., Subrizi F., Dobrijevic D., Hailes H. C., Ward J. M. (2021). Org. Biomol. Chem..

[cit20] Baierl A., Theorell A., Mackfeld U., Marquardt P., Hoffmann F., Moers S., Nöh K., Buchholz P. C. F., Pleiss J., Pohl M. (2018). ChemCatChem.

[cit21] Rios-Solis L., Halim M., Cázares A., Morris P., Ward J. M., Hailes H. C., Dalby P. A., Baganz F., Lye G. J. (2011). Biocatal. Biotransform..

[cit22] Strafford J., Payongsri P., Hibbert E. G., Morris P., Batth S. S., Steadman D., Smith M. E. B., Ward J. M., Hailes H. C., Dalby P. A. (2012). J. Biotechnol..

[cit23] Payongsri P., Steadman D., Hailes H. C., Dalby P. A. (2015). Enzyme Microb. Technol..

[cit24] Sprenger G. A., Schorken U., Wiegert T., Grolle S., de Graaf A. A., Taylor S. V., Begley T. P., Bringer-Meyer S., Sahm H. (1997). Proc. Natl. Acad. Sci. U. S. A..

