# Supplementary material for: Exploring the biocatalytic potential of wild-type split transketolases
Source: RSC Chem Biol. 2026 Oct 7. Online ahead of print. doi: 10.1039/d6cb00243a (PMC13663882; doi:10.1039/d6cb00243a)
Supplement: Supplementary file 1 [file CB-OLF-D6CB00243A-s001.pdf]

Supplementary Information (SI) for RSC Chemical Biology.
This journal is © The Royal Society of Chemistry

Supplementary Information

# Exploring the Biocatalytic Potential of Wild-Type 'Split' Transketolases

Alessia Tonoli, Silvia Anselmi, Yeke Ni, John M. Ward, Helen C. Hailes, Jack W. E. Jeffries*

**Production of split transketolases (split TKs).** Split TKs were expressed as recombinant proteins from synthetic two-gene operons, as previously described[1]. Protein expression, purification, and quantification were carried out following the previously reported procedures. The sequences and origins of the split TKs are also reported in the same study[1].

Table S1. pQR number and origin of the split TKs used in this study. For sequences derived from an organism, the organism is specified. For sequences derived from metagenomes, the putative organism assignment based on the closest homologues was specified, based on pBLAST E-values (last accessed June 2024).

| pQR number | Origin | Organism / Putative assignment |
|---|---|---|
| pQR2807 | Bacterium | *Fusobacterium nucleatum* subsp. *nucleatum* ATCC 25586 (N-terminal subunit) / *Fusobacterium* (multispecies, e.g. *F. nucleatum* subsp. *polymorphum* ATCC 10953) (chimera) (C-terminal subunit) |
| pQR2809 | Bacterium | *Thermotoga maritima* DSM 3109 / MSB8 |
| pQR2812 | Archaeon | *Methanocaldococcus jannaschii* DSM 2661 |
| pQR2815 | Archaeon | *Pyrococcus furiosus* DSM 3638 |
| pQR2823 | Salt-mine metagenome | *Patescibacteria group bacterium* (N-terminal subunit) / Candidatus *Kaiserbacteria bacterium* (C-terminal subunit) |
| pQR2825 | Salt-mine metagenome | *Planctomycetaceae bacterium* |
| pQR2827 | Salt-mine metagenome | *Planctomycetaceae bacterium* |
| pQR2828 | Salt-mine metagenome | Candidatus *Omnitrophota bacterium* |
| pQR2829 | Salt-mine metagenome | *Parcubacteria group bacterium* (N-terminal subunit) / Candidatus *Ryanbacteria bacterium* (C-terminal subunit) |
| pQR2830 | Salt-mine metagenome | *Parcubacteria group bacterium* (N-terminal subunit) / Candidatus *Paceibacterota bacterium* (C-terminal subunit) |
| pQR2834 | Bacterium | *Fusobacterium nucleatum* subsp. *nucleatum* ATCC 25586 |

**Production of reference full-length TKs.** Full-length TKs, *Eco*TK (TK from *E. coli*) and *Tma*TK (TK from *T. maritima*), were used as reference enzymes. *Eco*TK was expressed from a constitutive promoter[2] in *E. coli* DH5α cells by inoculating 200 mL LB medium (Sigma-Aldrich) in a 1 L flask with a 100X diluted overnight culture. Cultures were supplemented with 50 μg $mL^{-1}$ kanamycin (Bio Basic) and grown at 37 °C with shaking at 250 RPM for 16-20 hours. *Tma*TK was expressed from plasmid pQR1745[3,4] in LB medium (200 mL LB in a 1 L flask, supplemented with a 100X diluted overnight culture and 50 μg $mL^{-1}$ kanamycin) and induced with 1 mM IPTG (isopropyl β-D-1-thiogalactopyranoside, Sigma-Aldrich) at an $OD_{600}$ of 0.6. The culture was then incubated at 25 °C with shaking at 250 RPM for 16-20 hours. Purification and quantification of these full-length TKs were performed using the same procedures used for the split TKs.

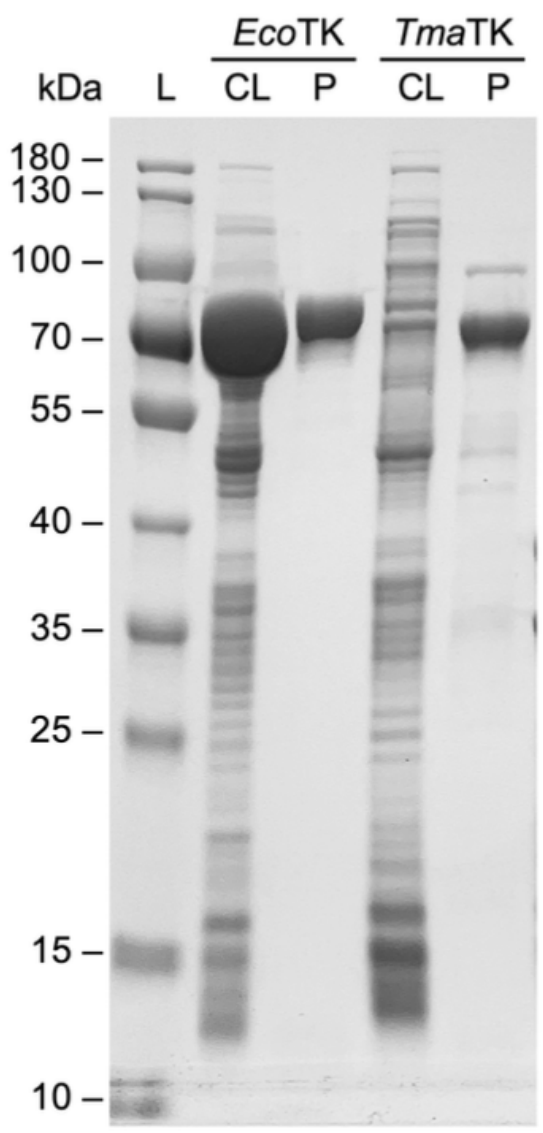


Figure S1. SDS-PAGE analysis of clarified lysate (CL) and purified (P) *Eco*TK and *Tma*TK. L = PageRuler Prestained Protein Ladder (Thermo Scientific).

**Biotransformations.** Biotransformations were performed in ThermoMixer C instruments equipped with Smartblock Thermoblocks and ThermoTop lids (Eppendorf). Reactions were carried out at 30 °C in 50 mM Tris-HCl buffer (pH 7.2), with the pH adjusted according to the reaction temperature using a correction factor of -0.028 pH units/°C, and contained 0.5 mg $mL^{-1}$ of purified enzyme (except *Tma*TK, 0.2 mg $mL^{-1}$), 2.4 mM ThDP (Sigma-Aldrich) and 9 mM $MgCl_2$ (Sigma-Aldrich). Prior to substrates addition, purified enzymes were incubated with the cofactors for at least 20 min at 20 °C with shaking at 700 RPM. End-point reactions were incubated for 20 hours, with shaking at 700 RPM for tube-based assays, and 1000 RPM for microplate assays. Microplates were sealed with aluminium sealing film (Starlab).

End-point assays for donor/acceptor combinations lithium β-hydroxypyruvate (HPA **1**, 32 mM, Sigma-Aldrich)/phenylacetaldehyde (PAA **2d**, 20 mM, Sigma-Aldrich) and pyruvate (PYR **4**, 40 mM, Sigma-Aldrich)/glycolaldehyde (GA **2a**, 20 mM, Sigma-Aldrich) were carried out in 96-well plates (150 µL final volume).

Time-courses assays for the determination of specific activities were performed in 1.5 mL tubes (700 µL final volume). Reactions were sampled and quenched at different time points, and product formation was quantified using the analytical methods described below. Specific activity was calculated from the slope of the initial linear portion of the product formation curve and normalised to the amount of enzyme used.

Assays for the determination of DMSO tolerance were stopped after 6 hours and contained different final concentrations of DMSO (from 10% to 40% (v/v), Sigma-Aldrich). PAA **2d** was prepared as a 10X stock solution in DMSO, resulting in a final DMSO concentration of 10% (v/v). Following incubation, reactions were stopped by dilution (10- or 20-fold) into water containing 0.1% (v/v) trifluoroacetic acid (TFA, Sigma-Aldrich). Samples were centrifuged for 20 minutes at maximum speed (>20,000 RCF) and the supernatants were analysed by HPLC. Product yields were calculated using calibration curves generated from product standards.

Reactions containing HPA **1** (32 mM) and the non-hydroxylated aliphatic acceptors propanal **2b** (20 mM) or pentanal **2c** (20 mM) were performed in 1.5 mL tubes (600 µL final volume). After incubation, samples were extracted with ethyl acetate (EtOAc, 3 x 300 µL), and the combined organic phase (750 µL) was collected and dried over $Na_2SO_4$. A 600 µL aliquot was transferred to a fresh tube and, after drying of EtOAc, samples were derivatised overnight at 20-22 °C with shaking at 700 RPM using 125 µL of derivatisation solution, consisting of 0.2 mg 4-(dimethylamino)pyridine (DMAP, Novabiochem), 20 µL pyridine (Sigma-Aldrich), 5 µL acetic anhydride ($Ac_2O$, Sigma-Aldrich), and 100 µL EtOAc per sample. Derivatised samples were diluted with 125 µL EtOAc, filtered through 0.22 µm PTFE filters into glass vials, and analysed by chiral GC to determine yield and optical purity. The derivatisation and analytical procedures were adapted from previously reported methods[5,6].

**HPLC analysis.** High-performance liquid chromatography (HPLC) analysis was carried out with Dionex UltiMate 3000 HPLC systems, equipped with an RS pump, an autosampler, a column compartment, an RS diode array detector, and a RefractoMax 521 refractive index (RI) detector (Thermo Fisher Scientific).
Reactions with HPA **1** and PAA **2d** were analysed using a ZORBAX Eclipse XDB-C18 column (4.6 x 150 mm, 5 µm) (Agilent Technologies) at 30 °C. The production of 1,3-dihydroxy-4-phenylbutan-2-one **3d** was detected and quantified via UV absorbance (DAD-3000RS) at 210 nm using the following gradient of eluent A ($H_2O$ with 0.1% (v/v) TFA) and eluent B (acetonitrile) at flow rate 1 mL $min^{-1}$: 0.0 min, 5% B; 1.5 min, 5% B; 2.0 min, 30% B; 13.5 min, 36% B; 14.5 min, 100% B; 15.5 min, 100% B; 16.0 min, 5% B; 17.0 min, 5% B. Retention times: **3d**, $t_R$ = 5.1 min.
Reactions with PYR **4** and GA **2a** were analysed using an Aminex HPX-87H column (Bio-Rad Laboratories) at 60 °C. An isocratic elution with $H_2O$ containing 0.1% (v/v) TFA at flow rate 0.6 mL $min^{-1}$ for up to 20 minutes was performed. Compounds were detected and quantified by RI detection with following retention times: erythrulose (ERY) **3a**, $t_R$ = 12.2 min; 3,4-dihydroxybutan-2-one (DHB) **5a**, $t_R$ = 14.5 min.

**GC analysis.** Gas chromatography (GC) analysis was carried out on a 7820A GC system (Agilent) equipped with a flame ionisation detector (FID) and a Supelco β-DEX 225 chiral column (30 m x 0.25 mm x 0.25 µm) (Sigma-Aldrich), using hydrogen as a carrier gas at flow rate of 6.5 mL $min^{-1}$. The temperature gradient for separation of (*R*)- and (*S*)-2-oxopentane-1,3-diyl diacetate (= diacetylated 1,3-dihydroxypentan-2-one **3b**) was as following: 40 °C, hold for 1 min, 2.5 °C $min^{-1}$ ramp to 140 °C, hold for 1 min, 20 °C $min^{-1}$ ramp to 220 °C, hold for 2 min (diacetylated (*R*)-**3b**, $t_R$ = 30.9 min; diacetylated (*S*)-**3b**, $t_R$ = 31.9 min, based on previous literature[5]). The temperature gradient for separation of (*R*)- and (*S*)-2-oxoheptane-1,3-diyl diacetate (= diacetylated 1,3-dihydroxyheptan-2-one **3c**) was as following: 80 °C, hold for 1 min, 3 °C $min^{-1}$ ramp to 118 °C, hold for 1 min, 0.4 °C $min^{-1}$ ramp to 122 °C, hold for 1 min, 3 °C $min^{-1}$ ramp to 160 °C, hold for 1 min, 20 °C $min^{-1}$ ramp to 220 °C, hold for 2 min (diacetylated (*R*)-**3c**, $t_R$ = 21.3 min; diacetylated (*S*)-**3c**, $t_R$ = 21.6 min; the assignment of the major (*S*)-isomer followed precedent with selectivity with **2b**).

**Synthesis of 3b-3d (TK biomimetic reaction).** The synthesis of dihydroxyketones **3b**-**3d** was achieved using the previously described TK biomimetic reaction [7-9]. N-methylmorpholine (2 mmol, 1.0 eq.) was dissolved in $H_2O$ (25 mL) in a round-bottom flask and the pH of the solution was adjusted to 8.0 (HCl). The required aldehyde (**2b**-**2d**, respectively; 3.0 mmol, 1.5 eq.) and HPA **1** (2.0 mmol, 1.0 eq.) were then added to the reaction mixture and left stirring overnight at RT. The reaction was monitored by thin layer chromatography (TLC), employing silica gel 60 F254 plates (Merck). Compounds of interest were detected under UV light, and then by staining with a potassium permanganate solution. Upon completion, the reaction was dried under vacuum and purified on Geduran silica gel 60 (40-63 µm). The purification of **3b** and **3c** was achieved using a mixture of 1:1 hexane/EtOAc. The purification of **3d** used to generate the calibration curve for the experiments shown in Figure 4 of the main text was performed on an Agilent 1260 Infinity system comprising a G2260A multisampler, two G1361A pumps, a G7165A muti-wavelength detector (MWD) and a G1364B fraction collector, equipped with a Supelco Discovery BIO Wide Pore C18-10 column (250 mm × 21.2 mm) with the UV detector at 1 = 214 nm, 2 = 254 nm, 3 = 280 nm; with a gradient elution using solvent A (water) and solvent B (MeCN) at a flow rate of 20 mL $min^{-1}$. The gradient was programmed as follows: 5% B (0-2 min), 5-95% B (2-10 min), 95% B (10-12 min), 95-5% B (12-12.1 min), and 5% B (12.1-15 min). In the case of **3d** used to generate the calibration curve for the experiments shown in Figure 1 of the main text, the achieved purity (44%, determined by qNMR) was deemed sufficient for running calibration.

**Peracetylation of 3b-3c.** The acetylation procedure was adapted from previously reported methods [5,6]. The dihydroxyketone (**3b** or **3c**) (1.33 mmol, 1.0 eq.) was dissolved in $CH_2Cl_2$ (5 mL), followed by the addition of DMAP (0.28 mmol, 0.2 eq.) and pyridine (3.99 mmol, 3.0 eq.). The reaction mixture was cooled to 0 °C before $Ac_2O$ (3.19 mmol, 2.4 eq.) was added while stirring. The mixture was then allowed to warm to room temperature and stirred for approximately 18 hours, with reaction progress monitored by TLC. The reaction was quenched with $H_2O$ (5 mL), and the product was extracted with $CH_2Cl_2$ (3 x 5 mL). The combined organic layers were washed with 0.1

M HCl (3 x 5 mL) and brine (5 mL), dried over $MgSO_4$, concentrated under vacuum, and purified by silica gel chromatography (hexane/EtOAc, 8:2).

**Biosynthesis of DHB 5a.** Product DHB **5a** was biosynthesised using purified split TKs pQR2823, pQR2829 and pQR2830. PYR **4** (100 mM) and GA **2a** (100 mM) were incubated with these split TKs (0.4 mg $mL^{-1}$) in 50 mM Tris-HCl buffer pH 7.5 containing 1 mM $MgCl_2$ and 0.1 mM ThDP in a final volume of 600 µL. Reactions were incubated at 30 °C with shaking at 750 RPM for 48 hours. The reactions were pooled, flash-frozen in liquid nitrogen and lyophilised with a BenchTop Pro freeze dryer (VirTis, SP Scientific). The dried residue was extracted with $CHCl_3$ (50 mL) followed by centrifugation (4000 g, 10 min, 4 °C). The supernatant was filtered through a cotton plug and the solvent removed on a rotary evaporator (30 °C) to dryness.

**NMR data.** $^{1}H$ and $^{13}C$ Nuclear Magnetic Resonance (NMR) spectra were acquired at 500 MHz on a Bruker Avance Neo 500 at 300 K using $CDCl_3$, $D_2O$ or DMSO-$d_6$. Chemical shifts (δ) are reported in ppm relative to the residual protic solvent. $^{1}H$ NMR multiplicities are denoted as s (singlet), d (doublet), t (triplet), m (multiplet), br s (broad singlet), or combinations thereof, with coupling constants (*J*) reported in Hz. $^{1}H$ and $^{13}C$ chemical shifts are reported to 0.01 and 0.1 ppm, respectively. Quantitative NMR (qNMR) was used to determine compound purity or concentration where required. Commercial ERY **3a** (Sigma-Aldrich) was analysed in $D_2O$ using dimethyl sulfone and maleic acid as internal standards [1]. **3d** used to generate the calibration curve for the experiments shown in Figure 1 of the main text was quantified in DMSO-$d_6$ using maleic acid as the internal standard. DHB **5a** was analysed in $CDCl_3$ using imidazole as the internal standard.

1,3-dihydroxypentan-2-one **3b**[5]

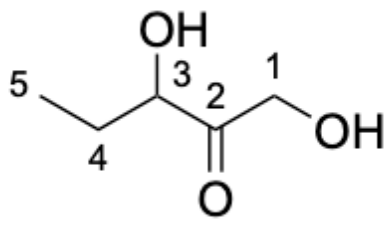

Yellow oil, 23% yield. $\delta_H$(500 MHz; $CDCl_3$) 4.48 (1 H, d, *J* 19.4 Hz, 1-*H*H), 4.38 (1 H, d, *J* 19.4 Hz, 1-H*H*), 4.27 (1 H, dd, *J* = 7.4, 4.3 Hz, 3-H), 2.98 (1 H, br s, O*H*), 1.92–1.81 (1 H, m, 1H, 4-*H*H), 1.63 (1H, dp, *J* = 14.6, 7.4 Hz, 4-H*H*), 0.98 (3H, t, *J* = 7.4 Hz, 5-$H_3$). $\delta_C$(126 MHz; $CDCl_3$) 212.1, 76.2, 66.0, 27.7, 9.2.

$^1$H NMR

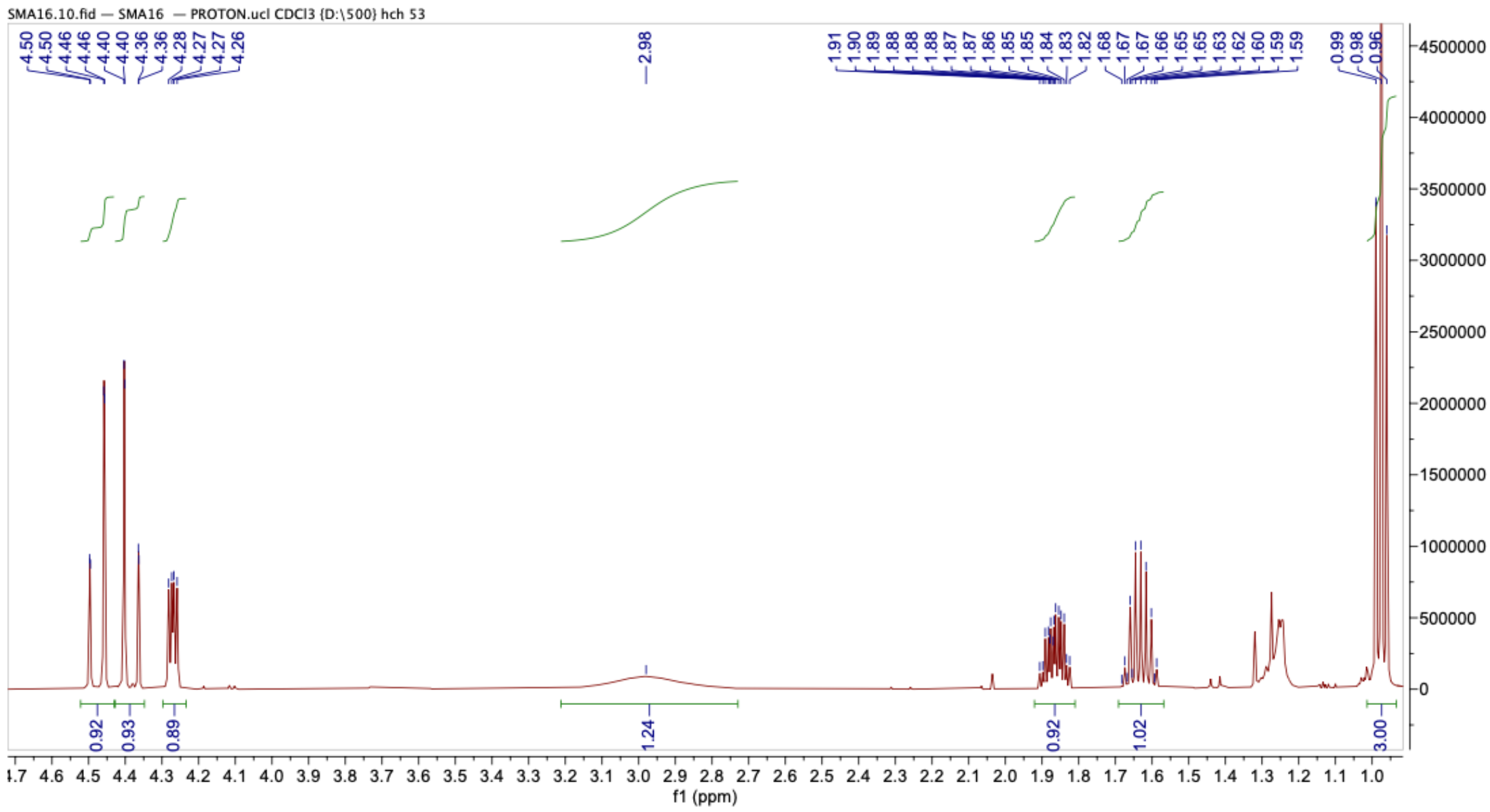

$^{13}$C NMR

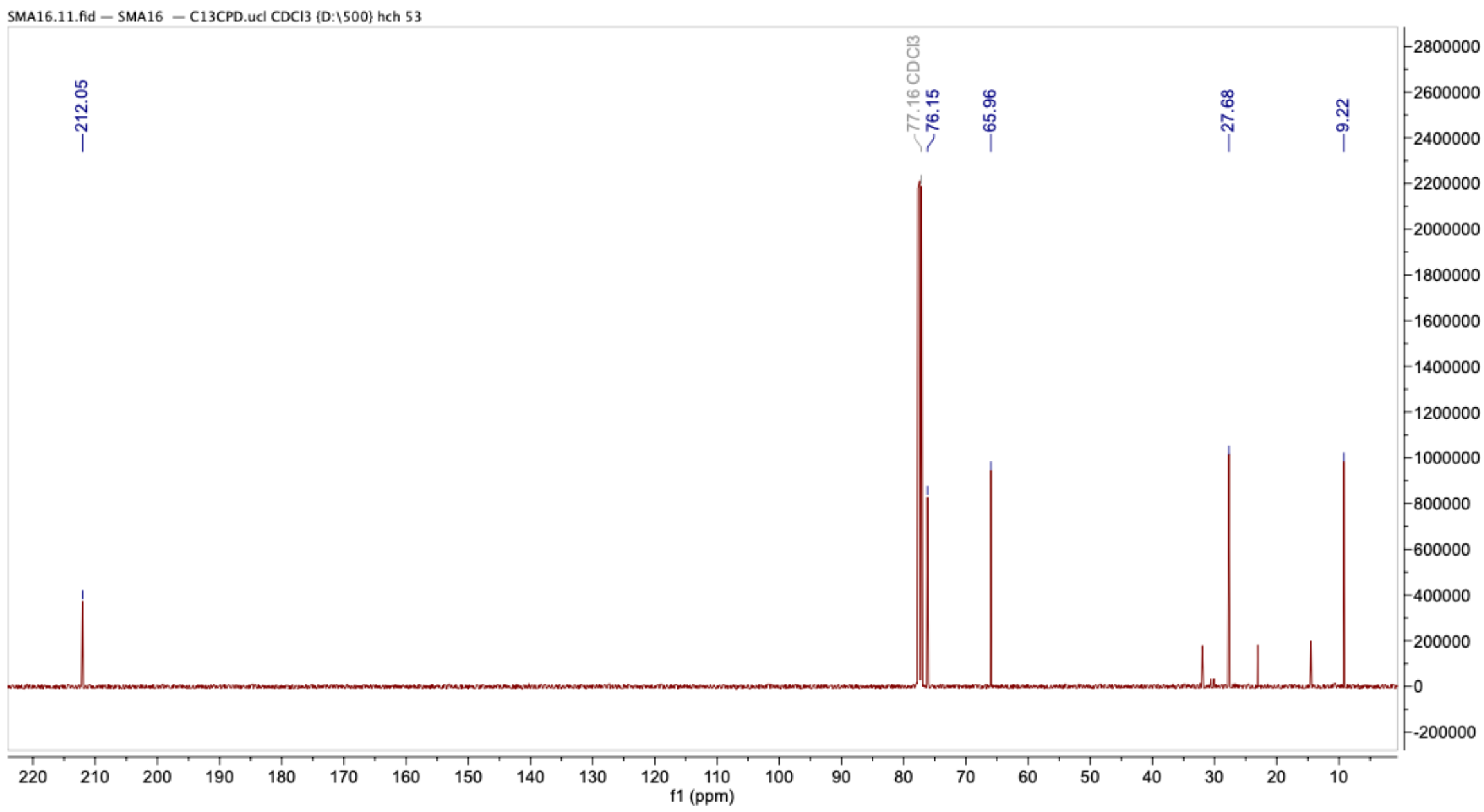

1,3-dihydroxyheptan-2-one **3c**[8]

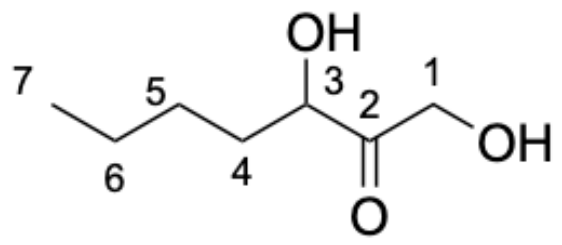


Yellow oil, 66% yield. $\delta_H$(500 MHz: $CDCl_3$) 4.49 (1 H, d, *J* = 19.4 Hz, 1-*H*H), 4.39 (1 H, d, *J* = 19.4 Hz, 1-H*H*), 4.31 (1 H, dd, *J* = 7.9, 3.9 Hz, 3-H), 1.86–1.74 (1 H, m, 4-*H*H), 1.67–1.53 (1 H, m, 4-H*H*), 1.51–1.29 (4 H, m, 5-$H_2$, 6-$H_2$), 0.96–0.87 (3 H, m, *3*H, 7-$H_3$). $\delta_C$(126 MHz; $CDCl_3$) 211.8, 75.0, 65.6, 34.1, 26.9, 22.6, 14.0.

$^{1}$H NMR

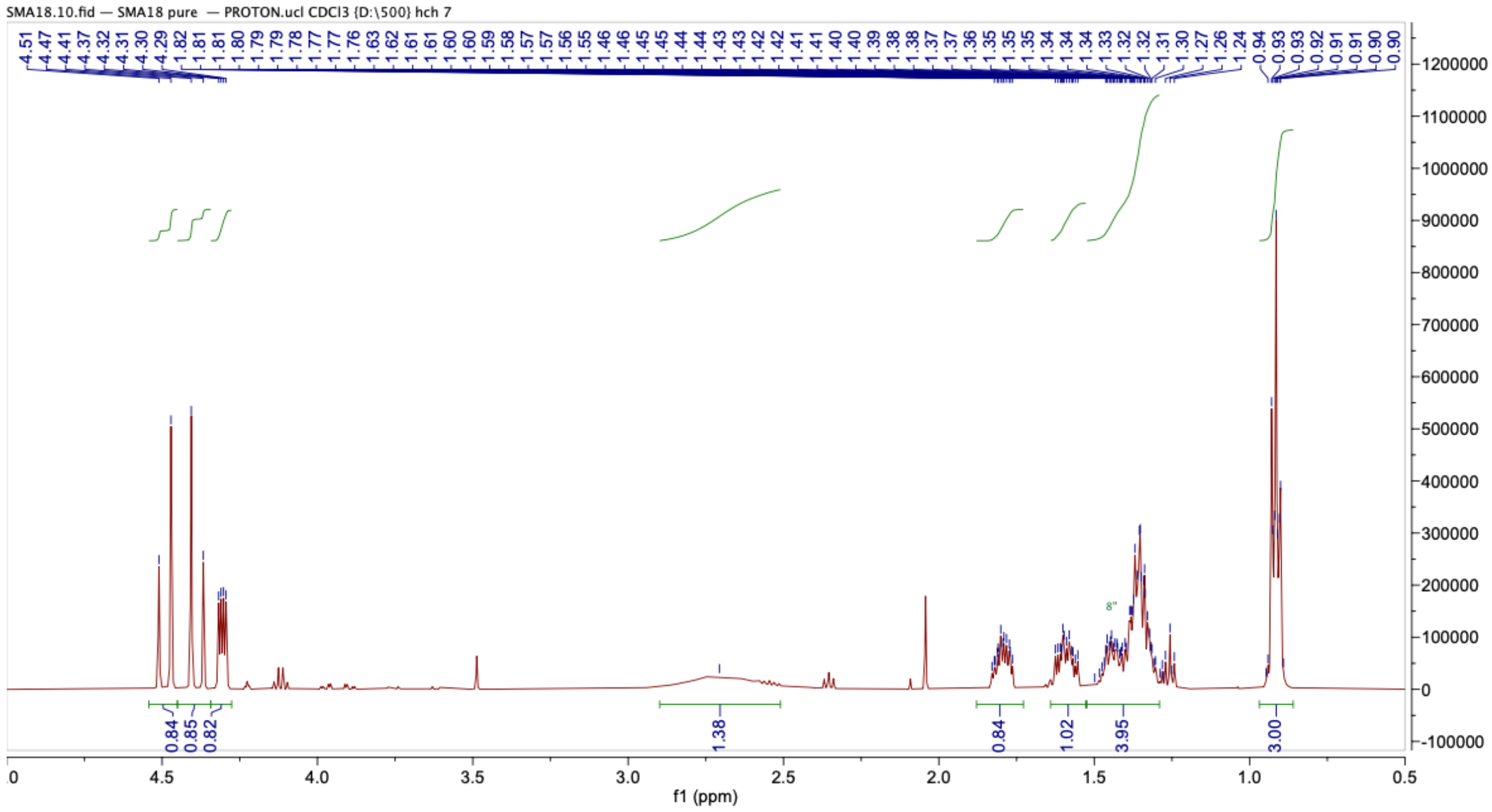


$^{13}$C NMR

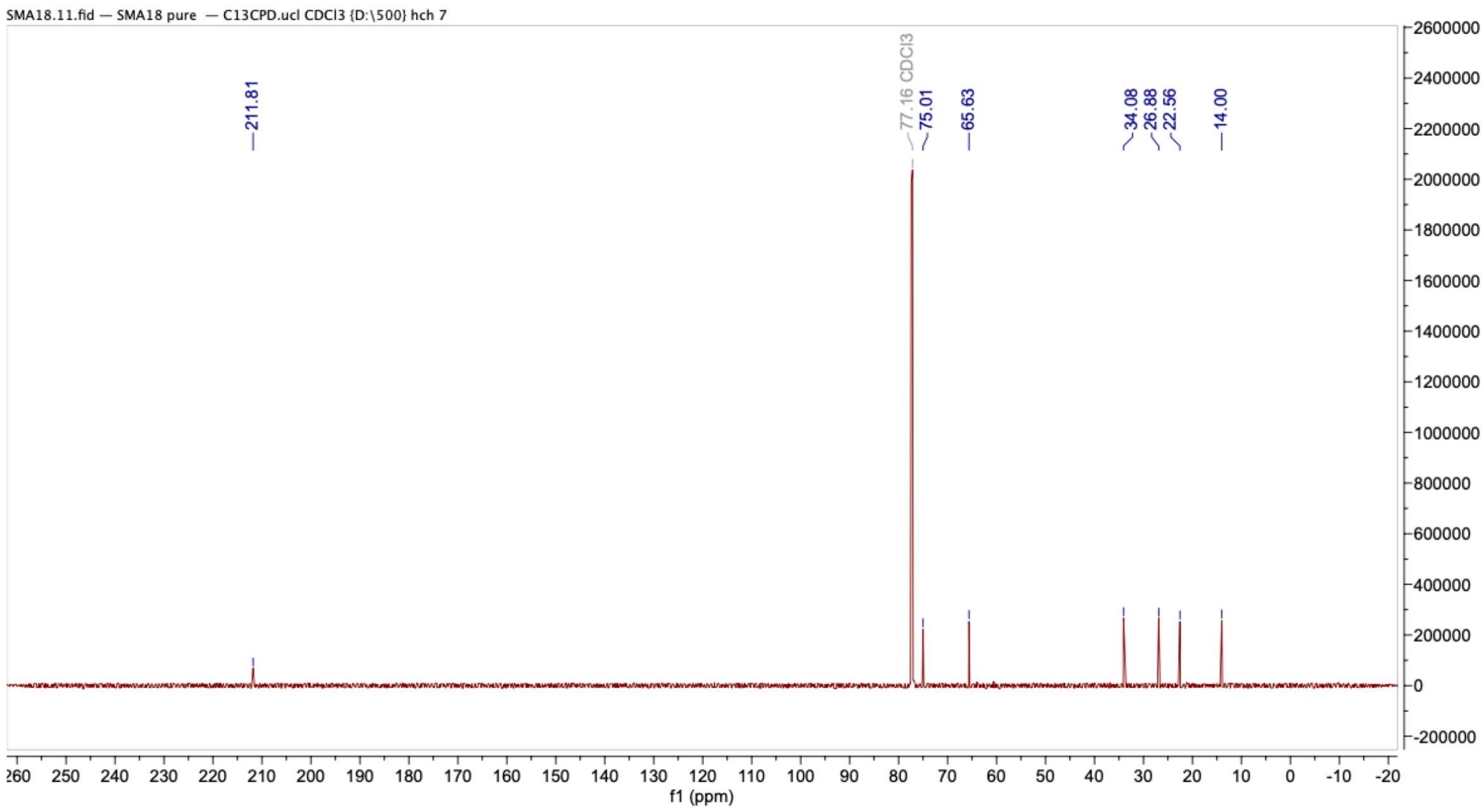

1,3-dihydroxy-4-phenylbutan-2-one **3d** [9]

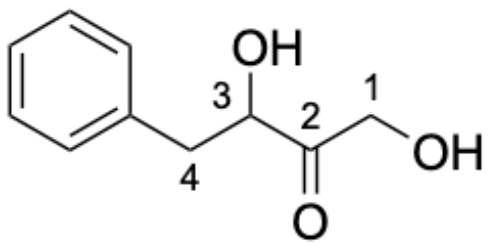


The NMR yield (44%) of **3d** used to generate the calibration curve for the experiments in Figure 1 of the main text was determined by $^{1}$H qNMR in DMSO_d$_6$, using maleic acid as standard. δ$_H$(500 MHz; CDCl$_3$) 7.36–7.20 (5 H, m, Ar-H), 4.47 (1 H, dd, *J* = 8.1, 4.6 Hz, 3-H), 4.42 (1 H, d, *J* = 19.8 Hz, 1-*H*H), 4.32 (1 H, d, *J* = 19.8 Hz, 1-H*H*), 3.12 (1 H, dd, *J* = 14.0, 4.6 Hz, 4-*H*H), 2.89 (1 H, dd, *J* = 14.0, 8.1 Hz, 4-H*H*). δ$_C$(126 MHz; CDCl$_3$) 211.3, 135.7, 129.4, 129.0, 127.4, 76.1, 66.4, 40.6.

$^{1}$H NMR

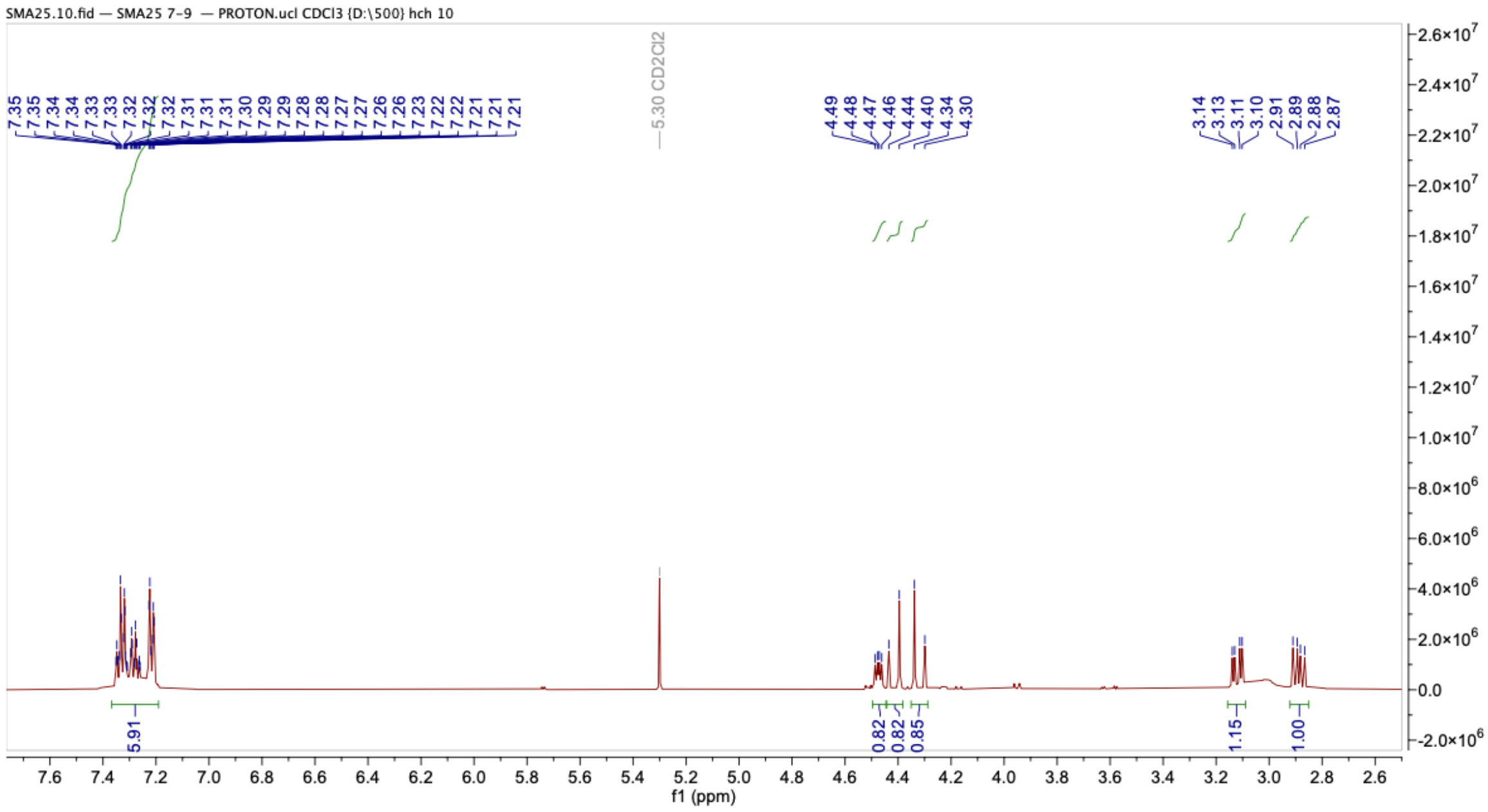


$^{13}$C NMR

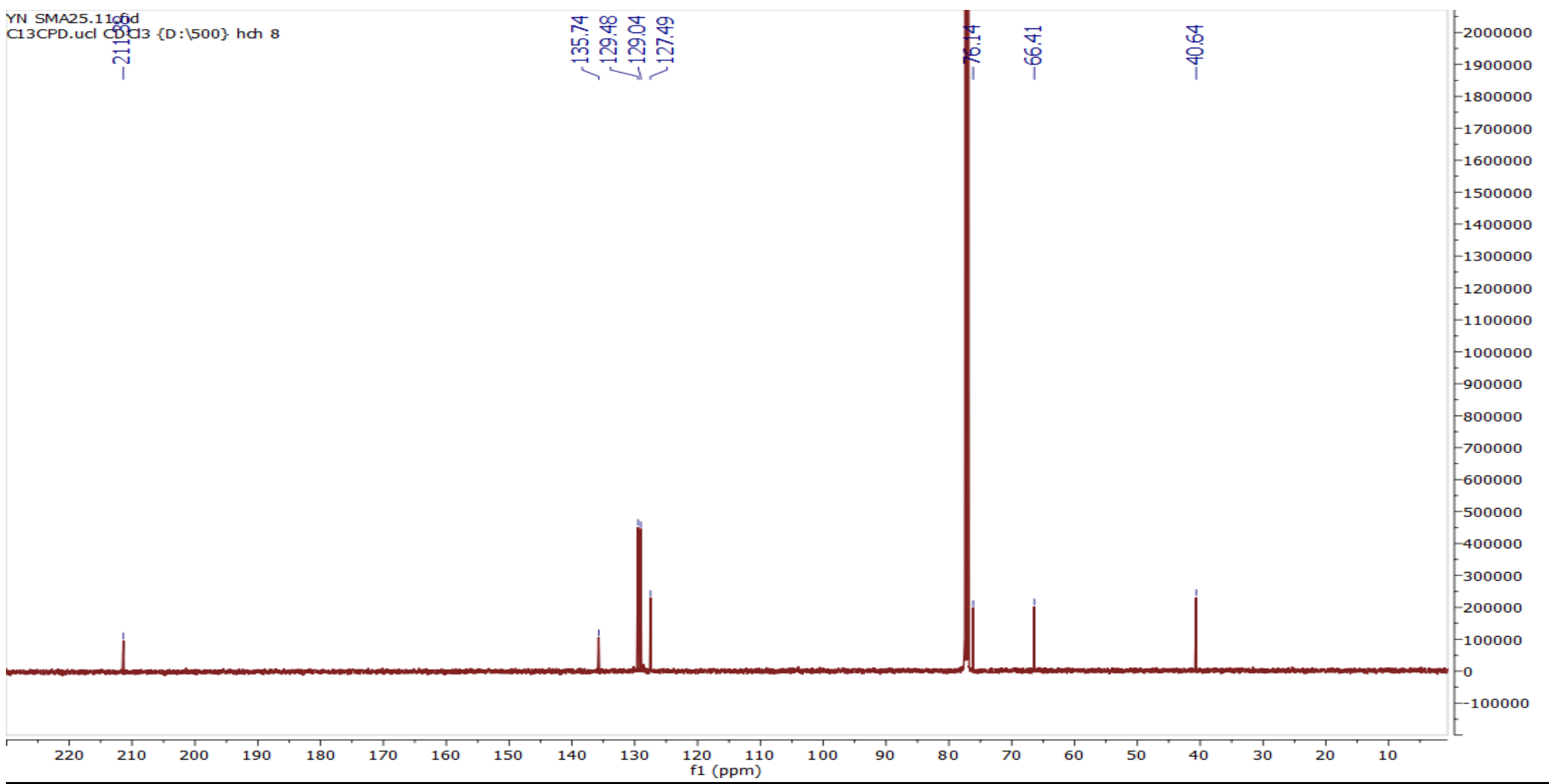

3,4-dihydroxybutan-2-one (DHB) **5a** [10]

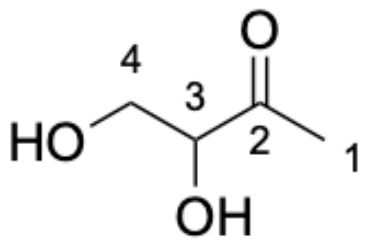


The NMR yield (40%) of **5a** was determined by $^{1}$H qNMR in $CDCl_3$, using imidazole as standard. $\delta_H$(500 MHz; $CDCl_3$) 4.23 (1 H, t, *J* = 3.4 Hz, 3-H), 3.95 (1 H, dd, *J* = 12.1, 3.4 Hz, 4-*H*H), 3.90 (1 H, dd, *J* = 12.1, 3.4 Hz, 4-H*H*), 2.27 (3 H, s, 1-$H_3$). $\delta_C$(126 MHz; $CDCl_3$) 208.2, 78.3, 63.6, 25.3.

$^{1}$H NMR

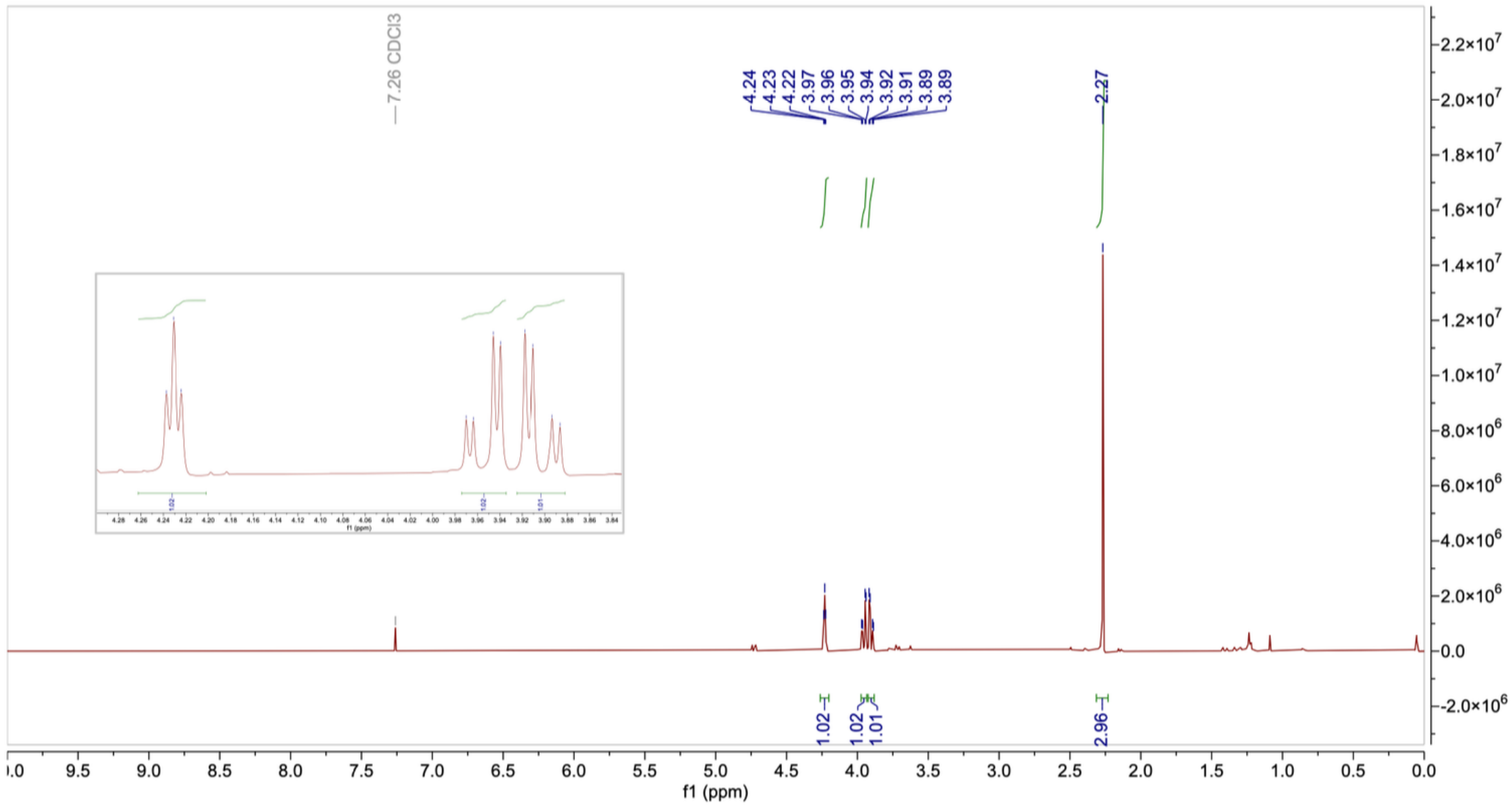


$^{13}$C NMR

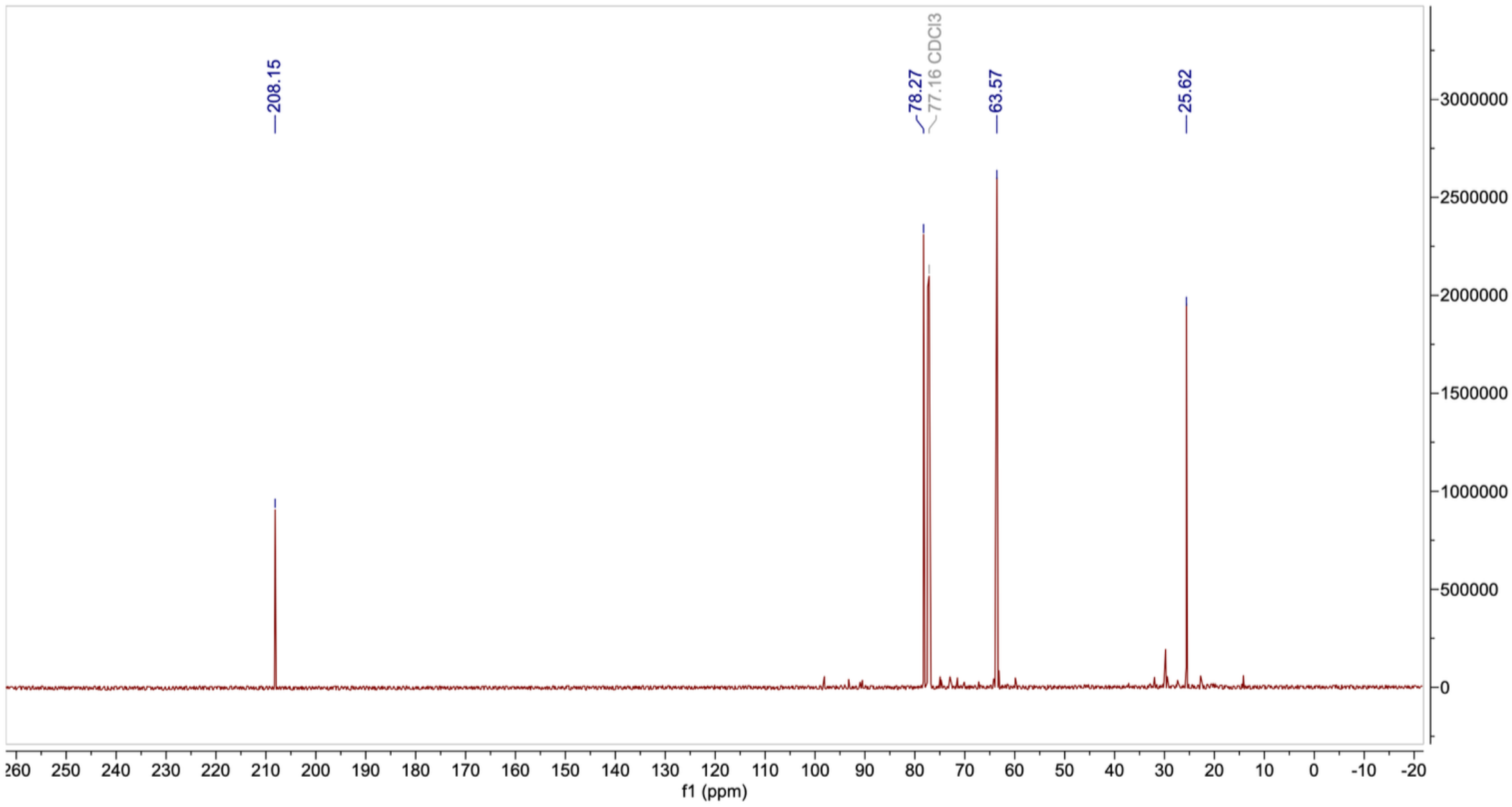

2-oxopentane-1,3-diyl diacetate (= diacetylated **3b**) [5]

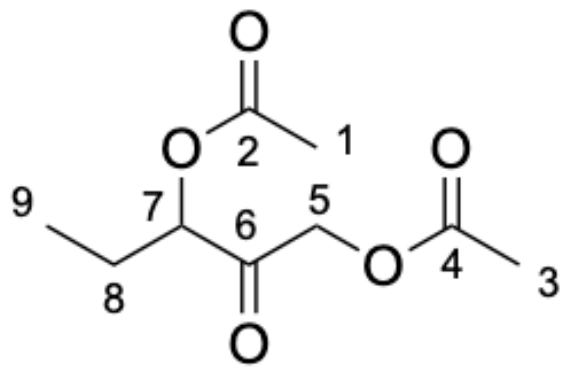

Synthesised from **3b**. Pale yellow oil, 68% yield. $\delta_H$(500 MHz; $CDCl_3$) 5.07 (1 H, dd, *J* = 7.6, 4.9 Hz, 7-H), 4.83 (1 H, d, *J* = 17.2 Hz, 5-*H*H), 4.77 (1 H, d, *J* = 17.2 Hz, 5-H*H*), 2.16 (3 H, s, $COCH_3$), 2.15 (3 H, s, $COCH_3$), 1.96–1.82 (2 H, m, 8-$H_2$), 0.99 (3 H, t, *J* = 7.4 Hz, 9-$H_3$). $\delta_C$(126 MHz; $CDCl_3$) 200.9, 170.6, 170.3, 77.7, 66.3, 24.2, 20.7, 20.6, 9.4.

$^1$H NMR

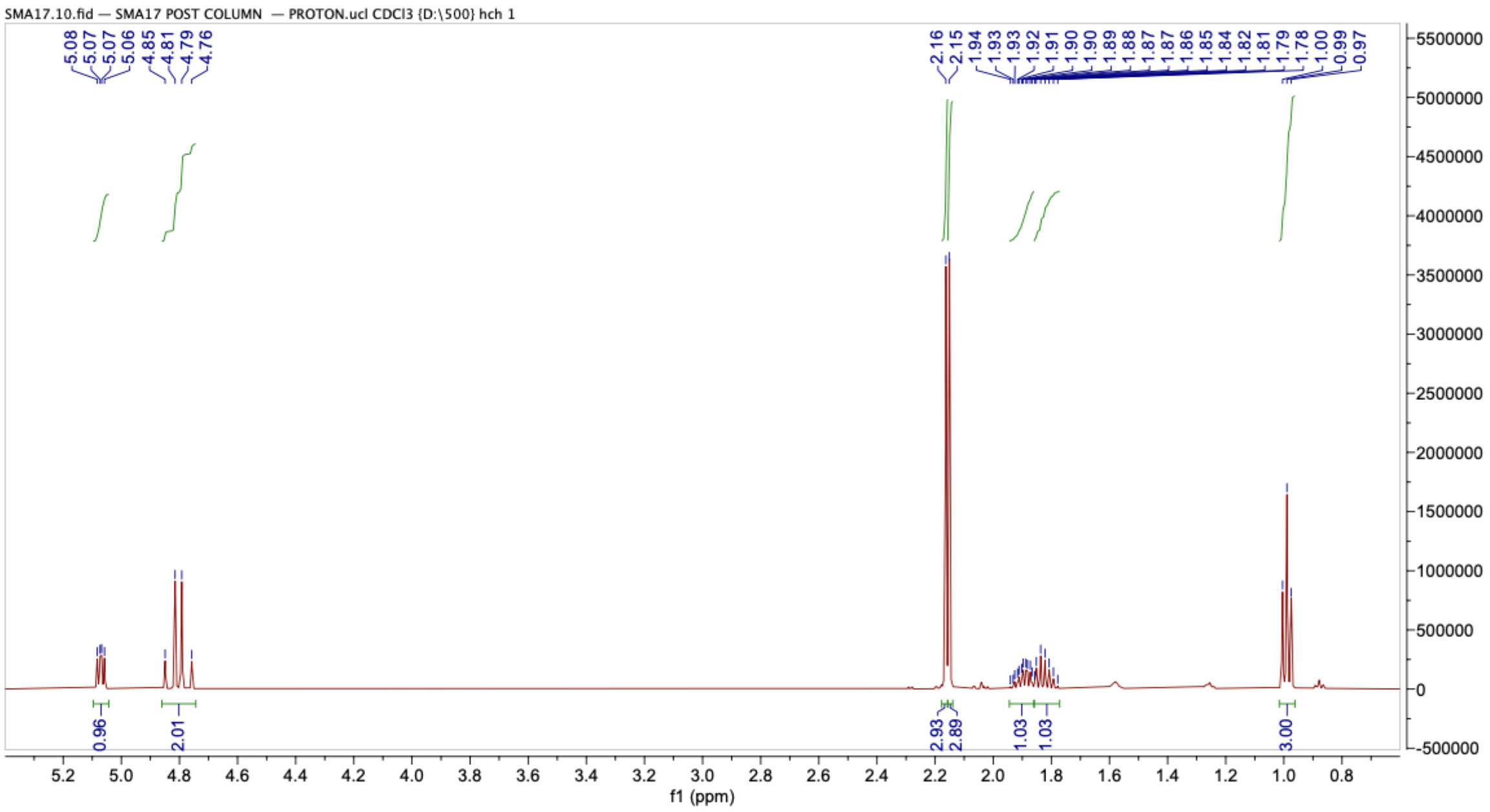


$^{13}$C NMR

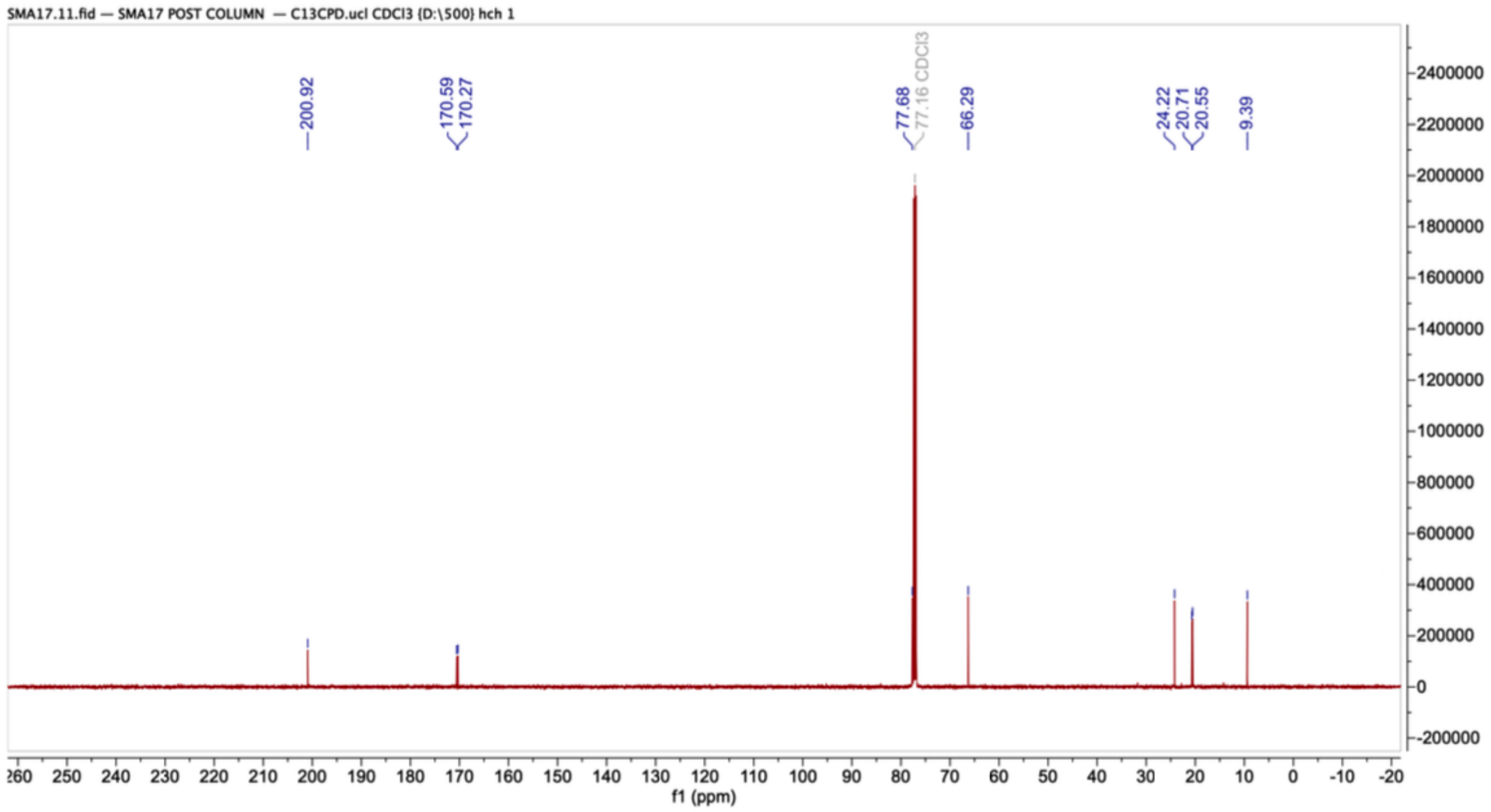

2-oxoheptane-1,3-diyl diacetate (= diacetylated **3c**)

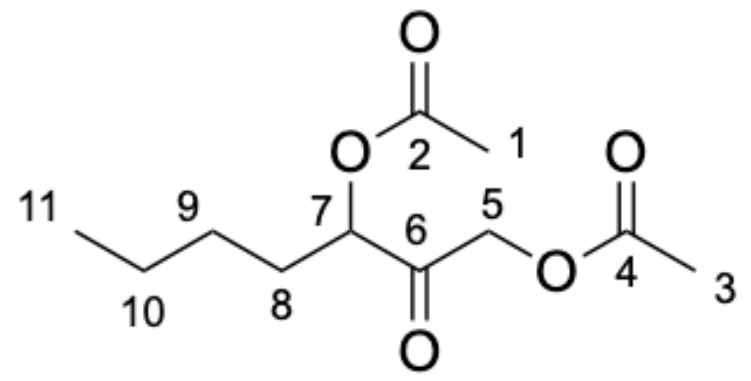


Synthesised from **3c**. Pale yellow oil, 97% yield. $\delta_H$(500 MHz; $CDCl_3$) 5.11 (1 H, dd, *J* = 8.1, 4.7 Hz, 7-H), 4.83 (1 H, d, *J* = 17.2 Hz, 5-*H*H), 4.77 (1 H, d, *J* = 17.2 Hz, 5-H*H*), 2.16 (3 H, s, $COCH_3$), 2.14 (3 H, s, $COCH_3$), 1.88–1.72 (2 H, m, 8-$H_2$), 1.44–1.31 (4 H, m, 9-$H_2$, 10-$H_2$), 0.91 (3 H, t, *J* = 7.1 Hz, 11-$H_3$). $\delta_C$(126 MHz; $CDCl_3$) 201.0, 170.6, 170.3, 76.7, 66.2, 30.5, 27.2, 22.4, 13.9.

$^1$H NMR

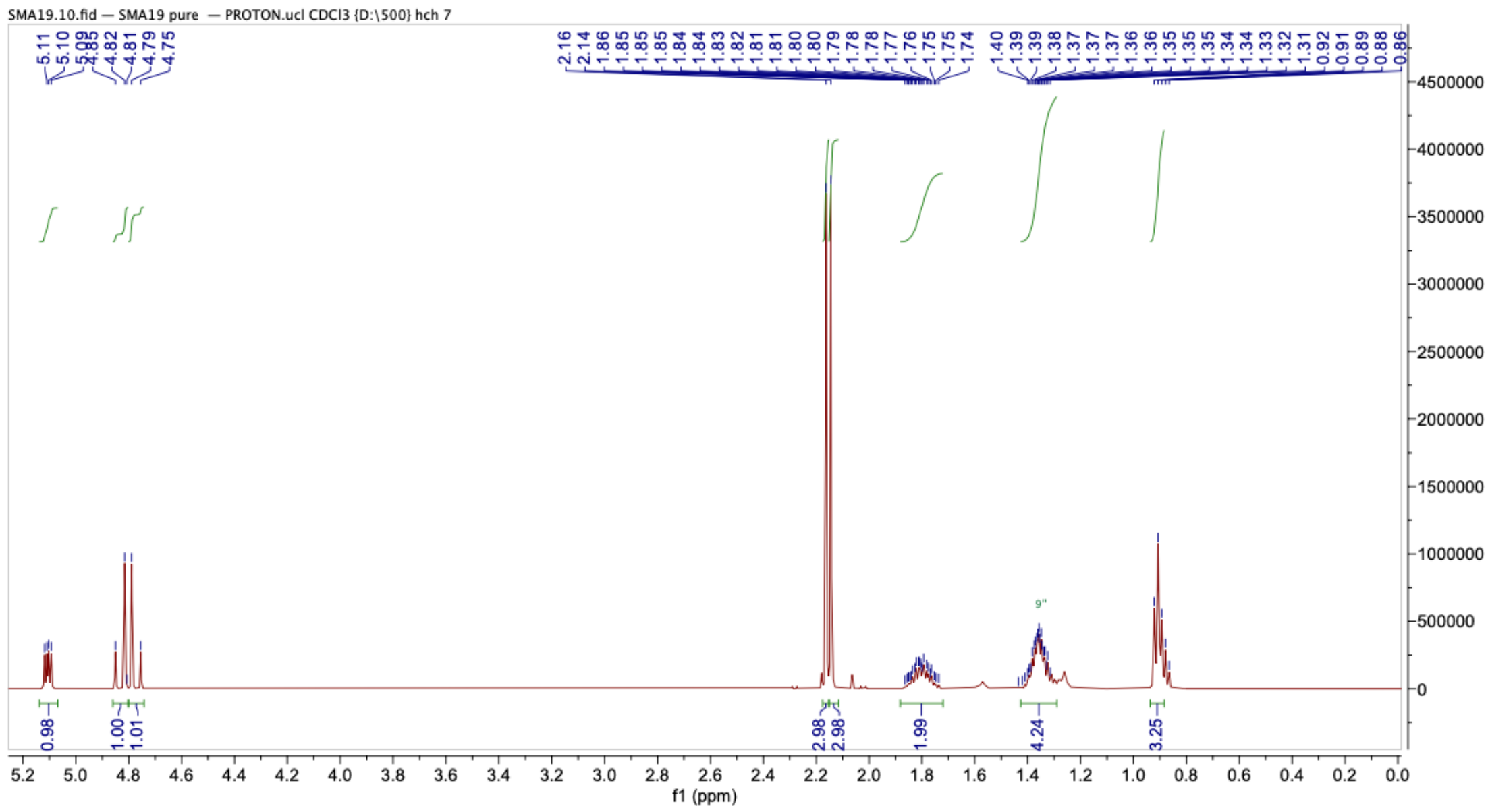


$^{13}$C NMR

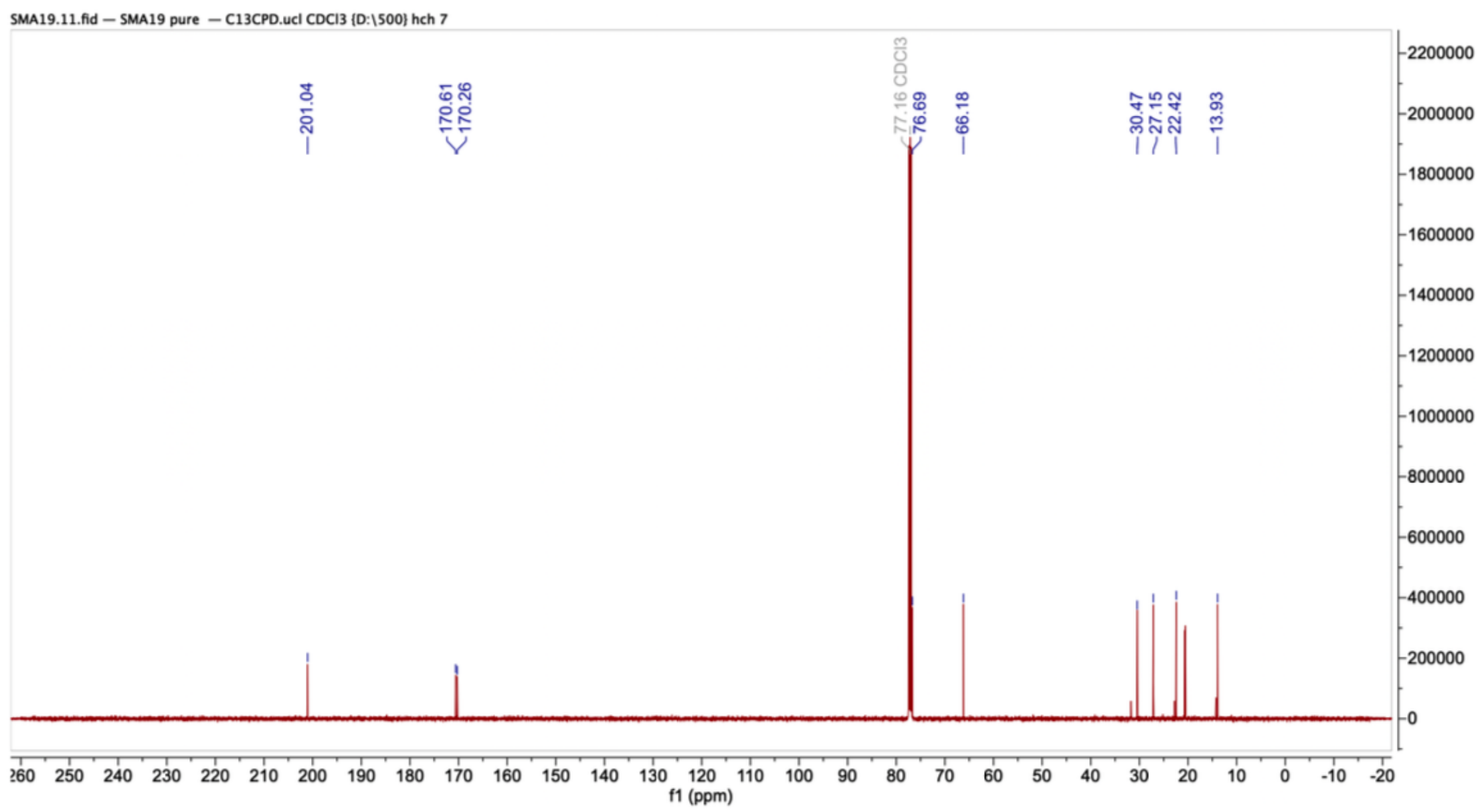

## HPLC/GC traces

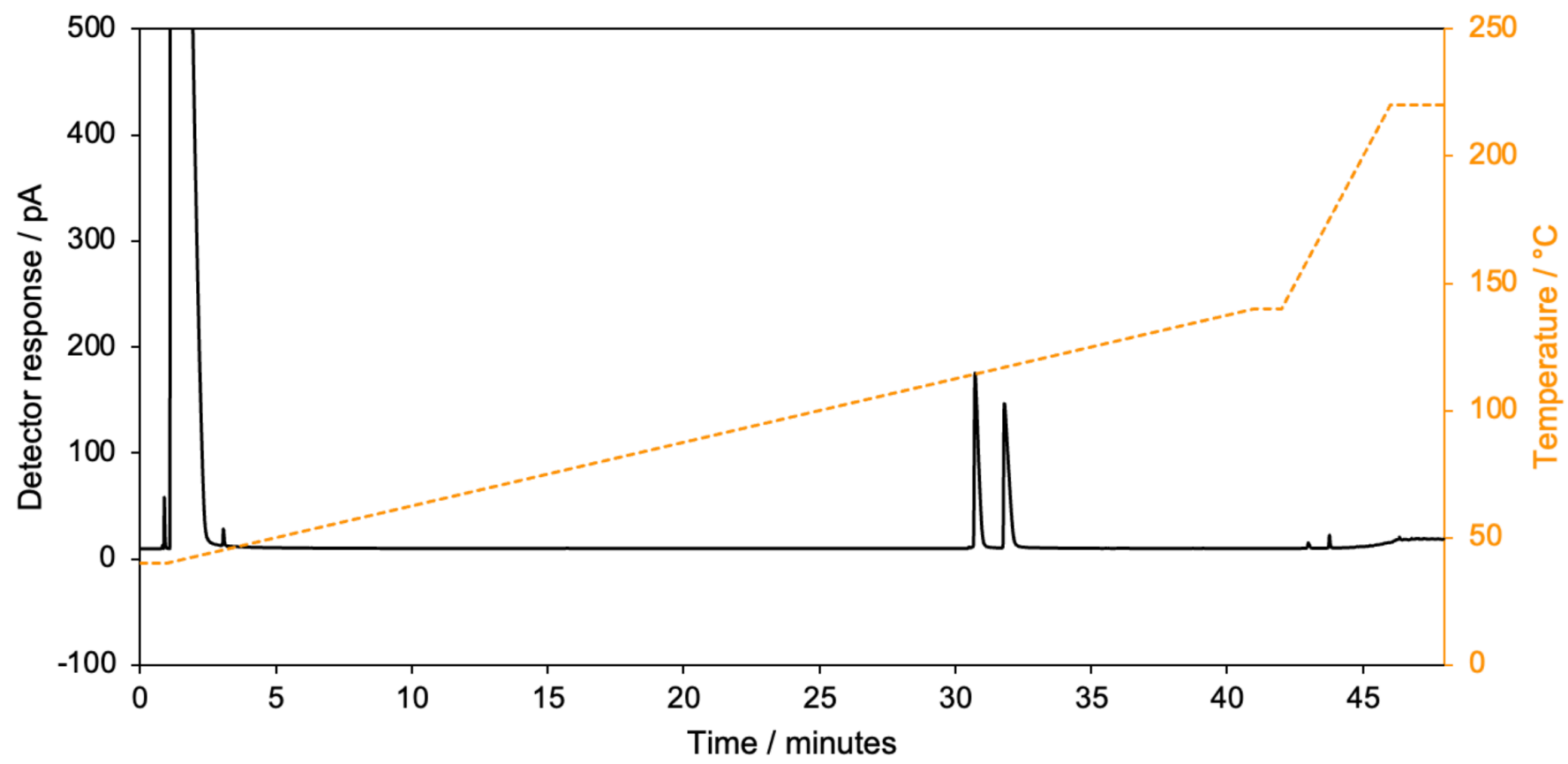


Figure S2. Chiral GC-FID trace of racemic diacetylated **3b**. Diacetylated (*R*)-**3b**, $t_R$ = 30.9 min; diacetylated (*S*)-**3b**, $t_R$ = 31.9 min.

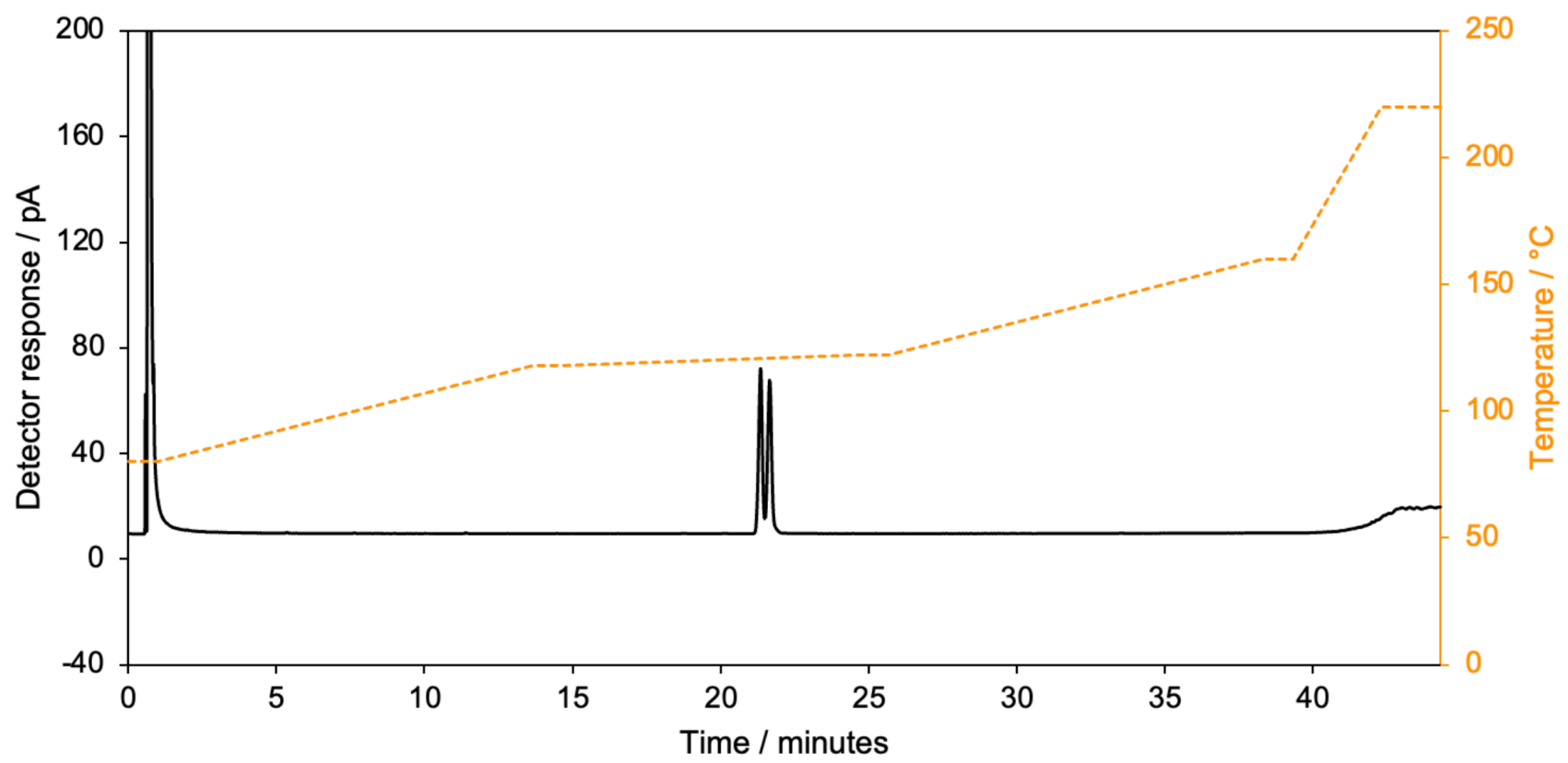


Figure S3. Chiral GC-FID trace of racemic diacetylated **3c**. Diacetylated (*R*)-**3c**, $t_R$ = 21.3 min; diacetylated (*S*)-**3c**, $t_R$ = 21.6 min

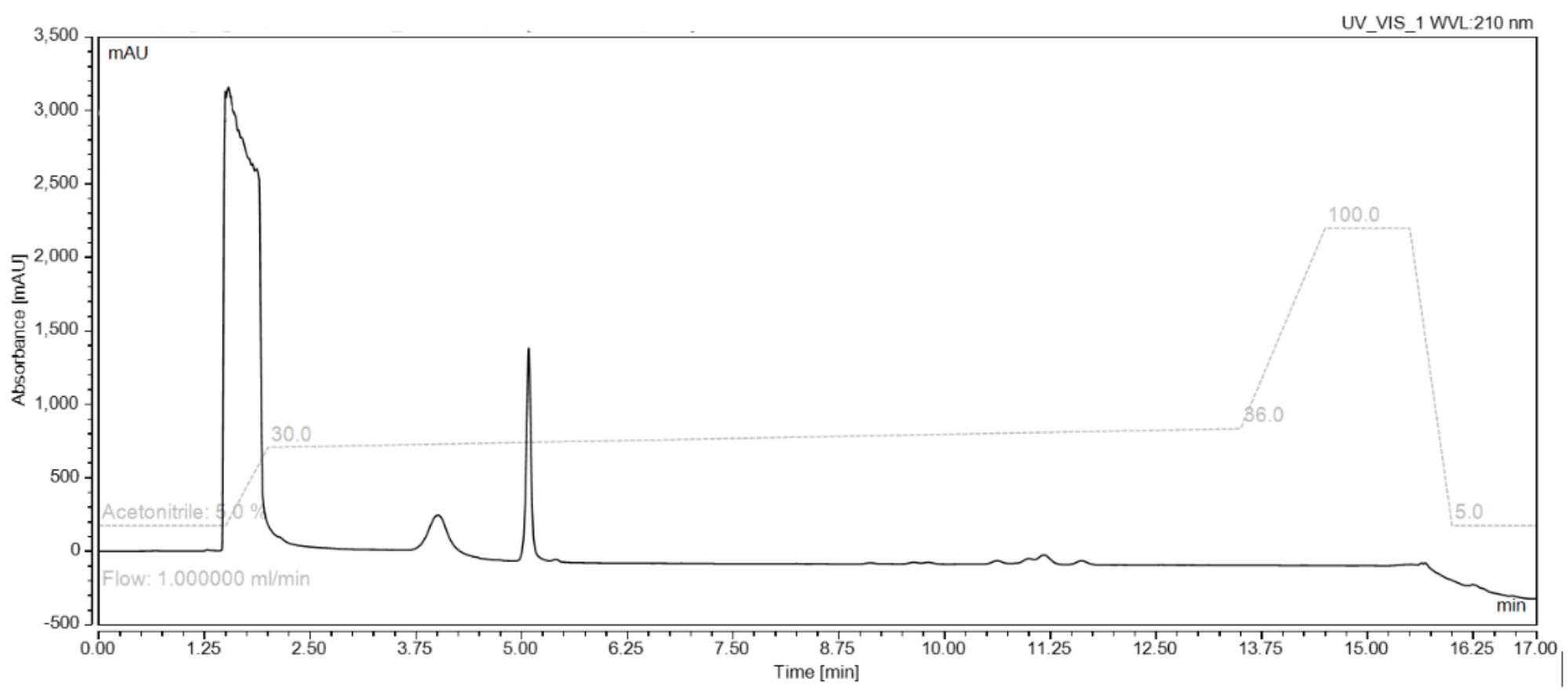


Figure S4. HPLC trace of **3d**, $t_R$ = 5.1 min.

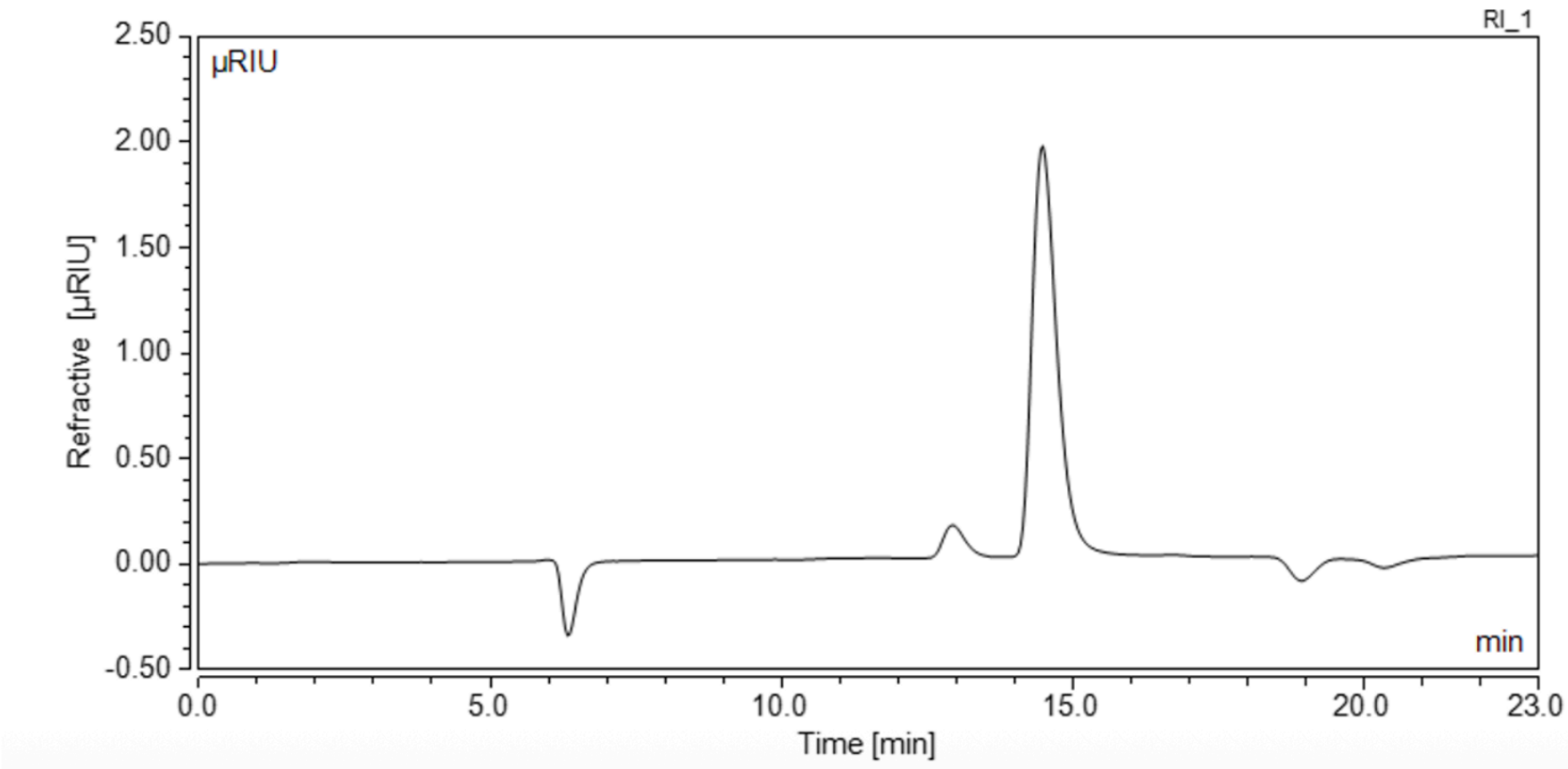


Figure S5. HPLC trace of DHB **5a**, $t_R$ = 14.5 min.

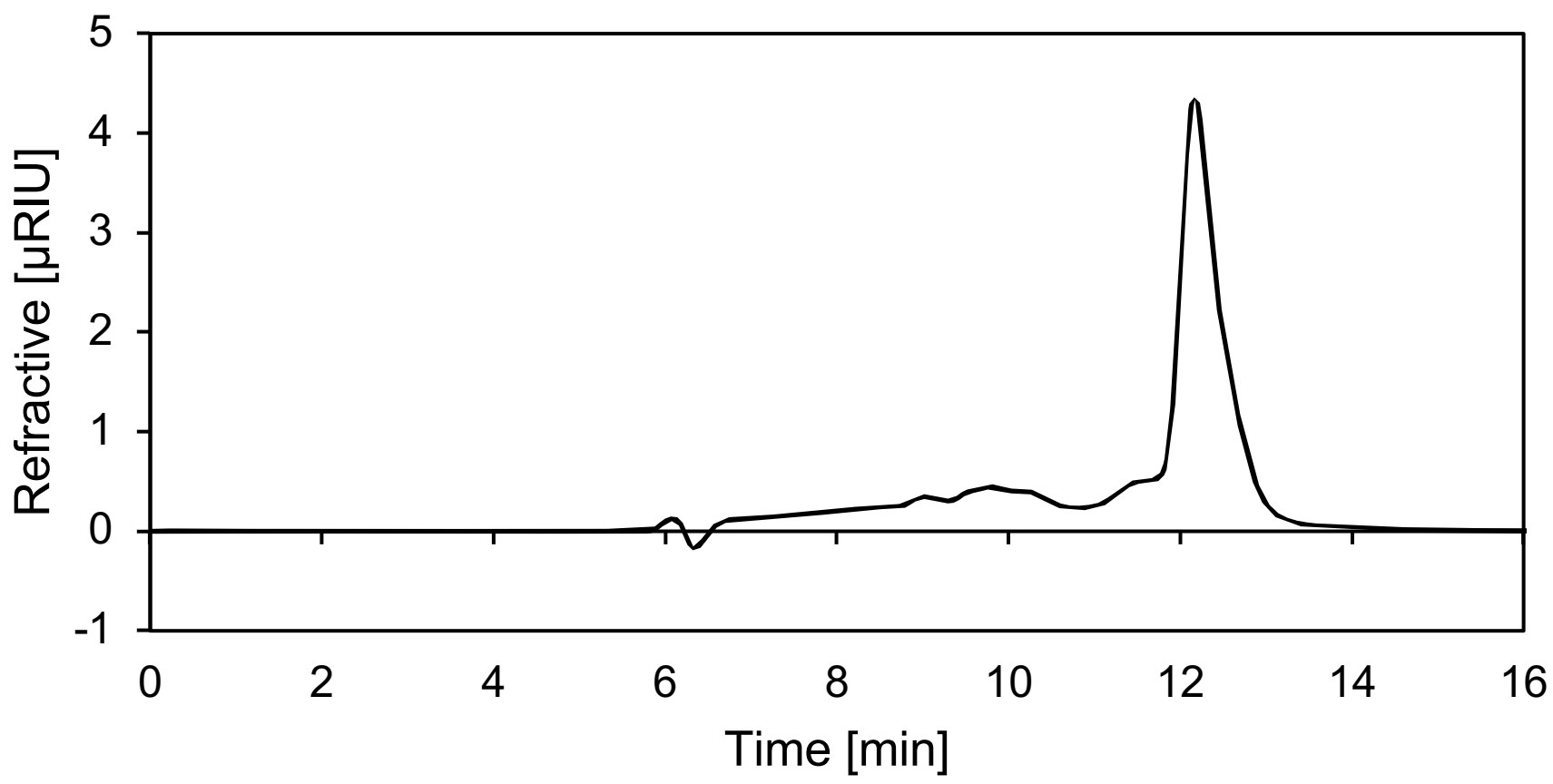


Figure S6. HPLC trace of ERY **3a**, $t_R$ = 12.2 min.

**HPLC/GC calibration curves**

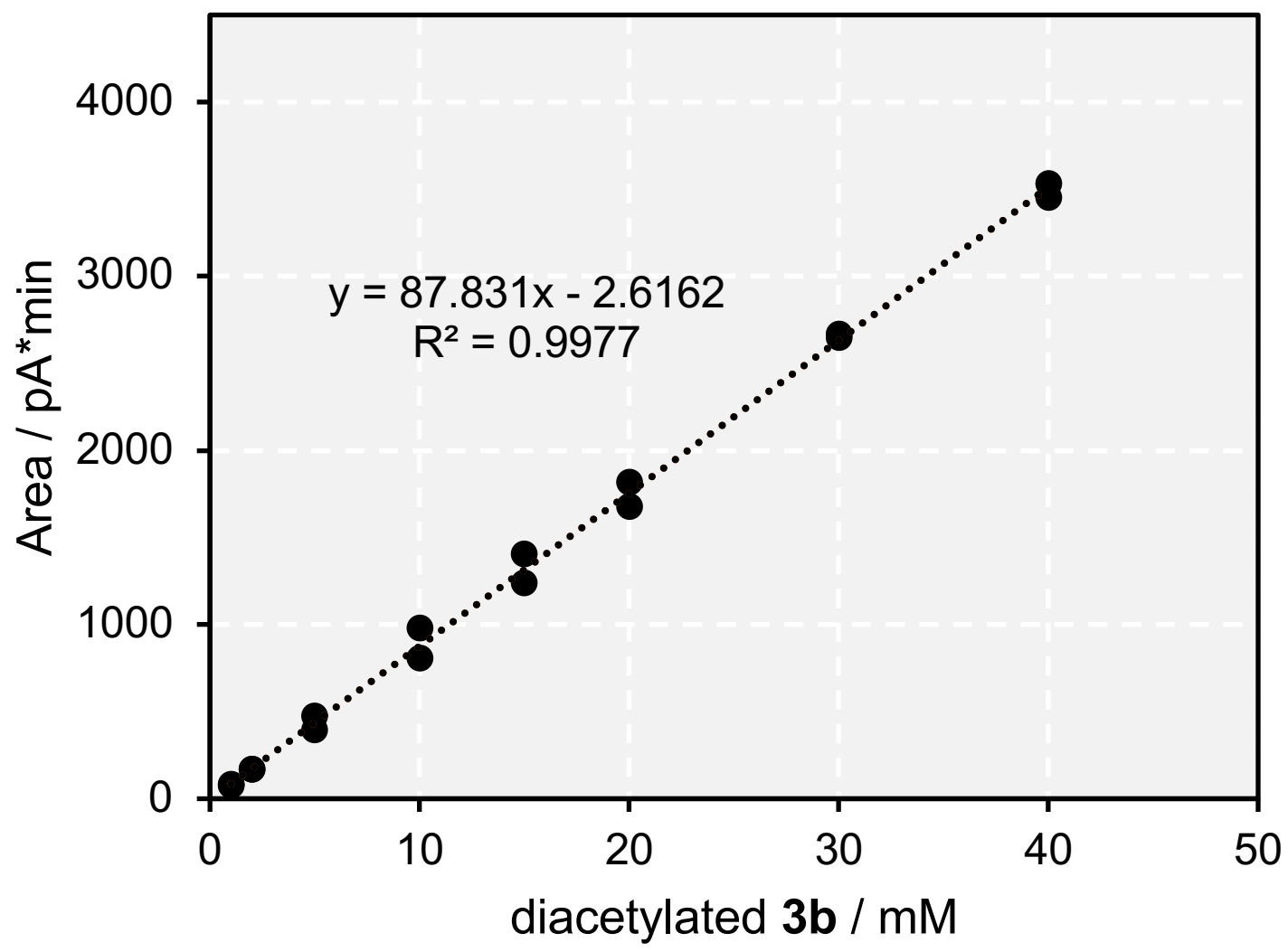


Figure S7. Calibration curve of diacetylated **3b** (GC-FID).

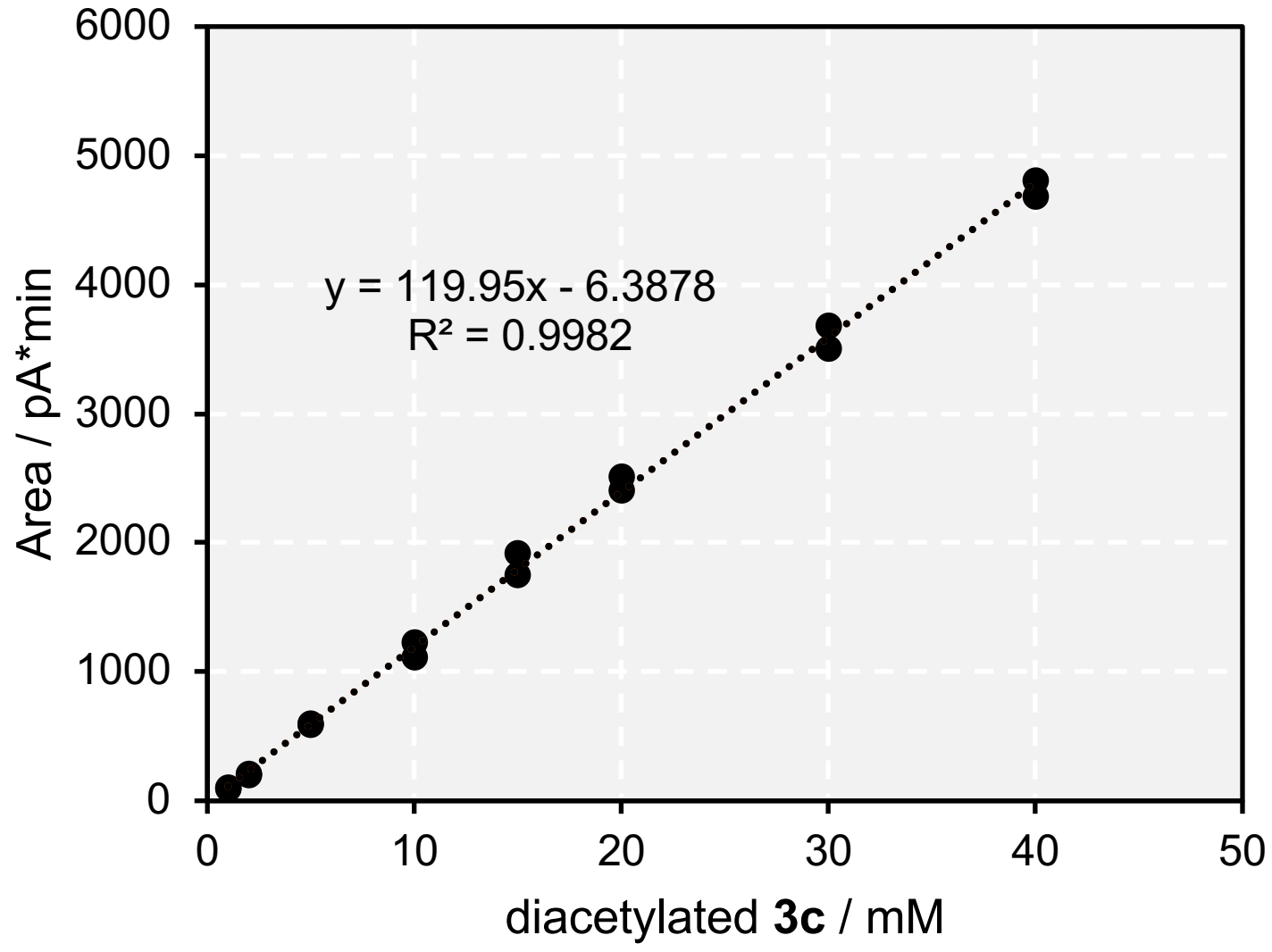


Figure S8. Calibration curve of diacetylated **3c** (GC-FID).

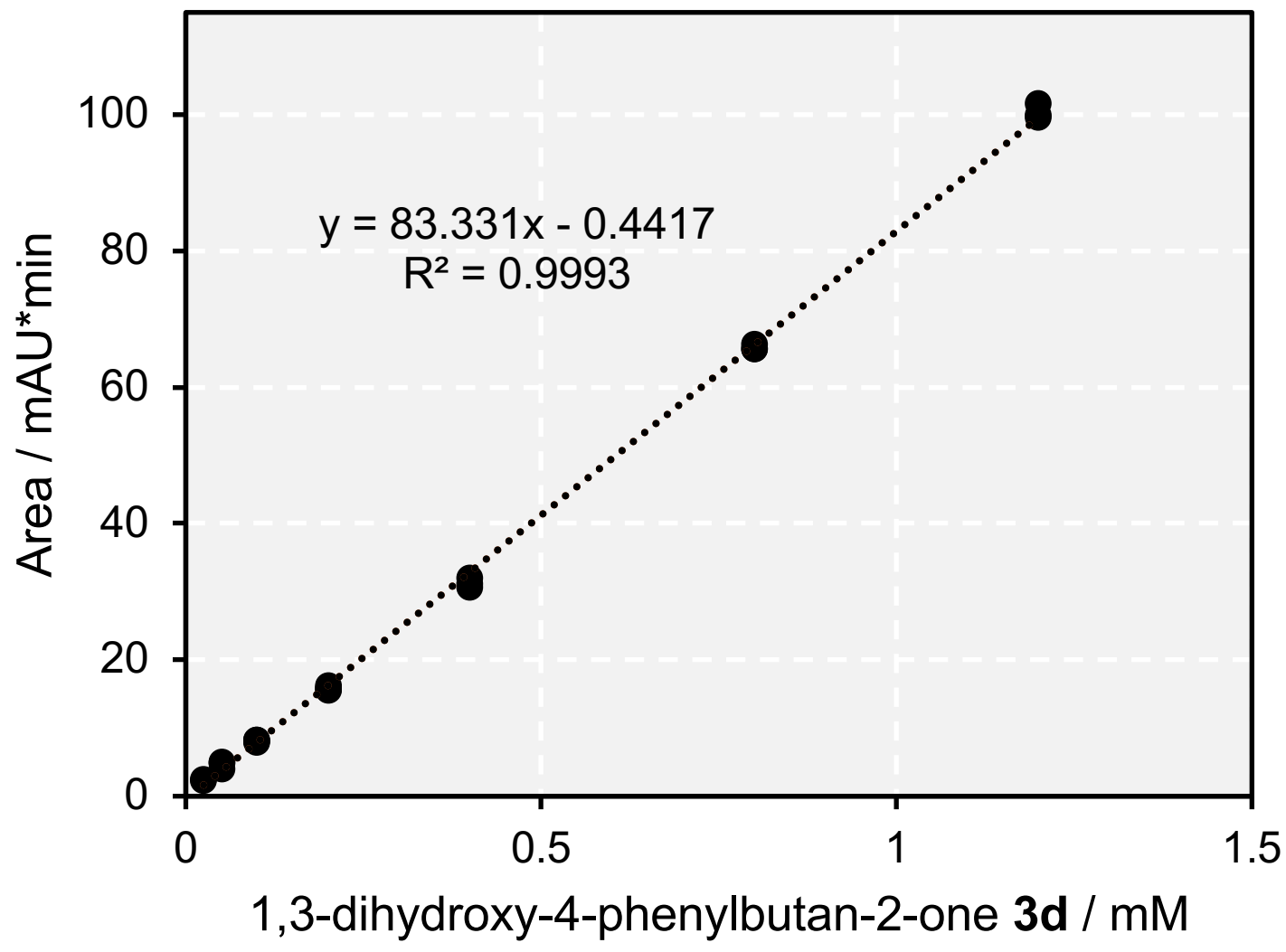


Figure S9. Calibration curve of 1,3-dihydroxy-4-phenylbutan-2-one **3d** (HPLC-UV 210 nm) used for HPLC yield calculations for the experiments shown in Figure 1 of the main text.

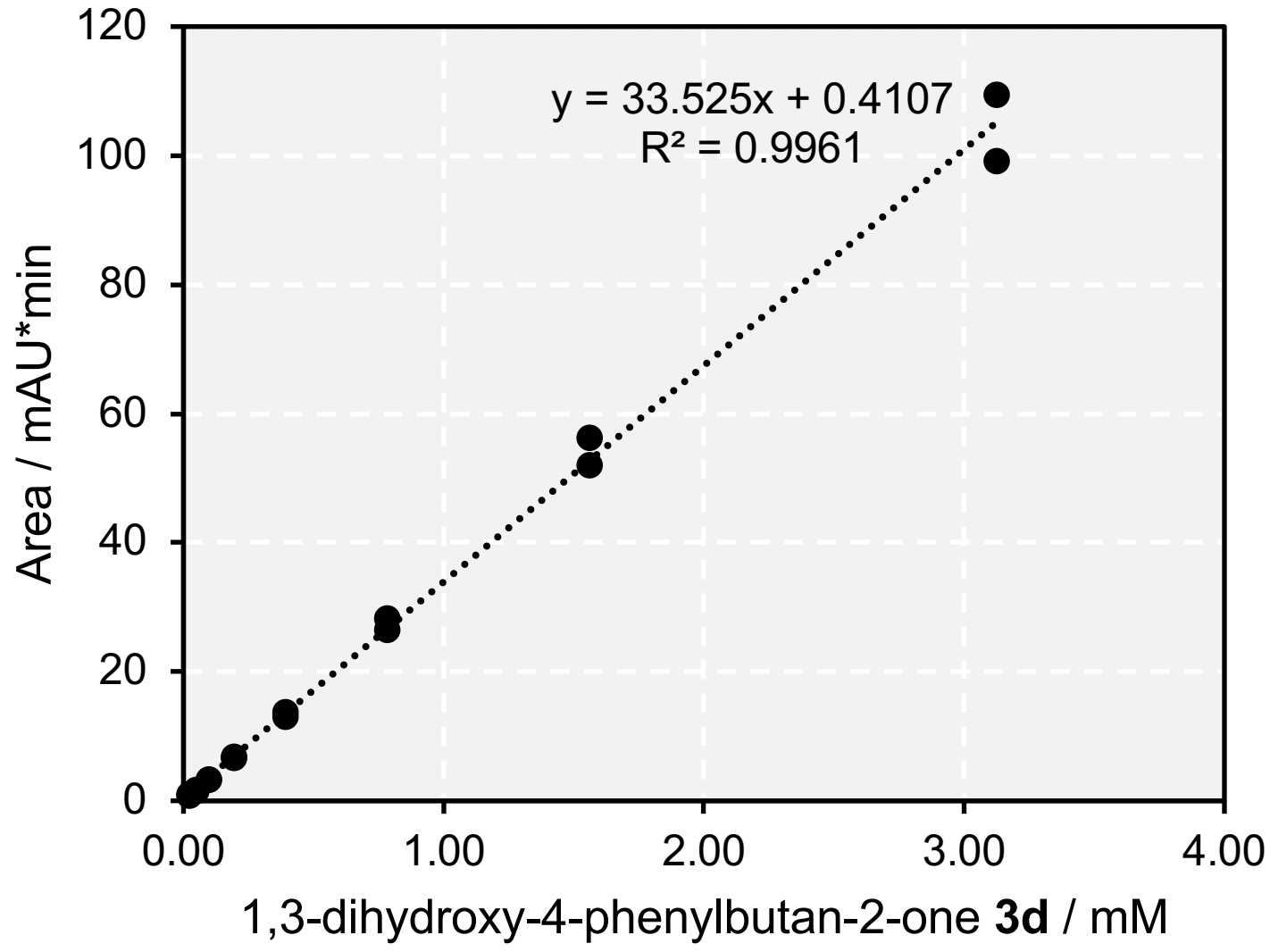


Figure S10. Calibration curve of 1,3-dihydroxy-4-phenylbutan-2-one **3d** (HPLC-UV 210 nm) used for HPLC yield calculations for the experiments shown in Figure 4 of the main text.

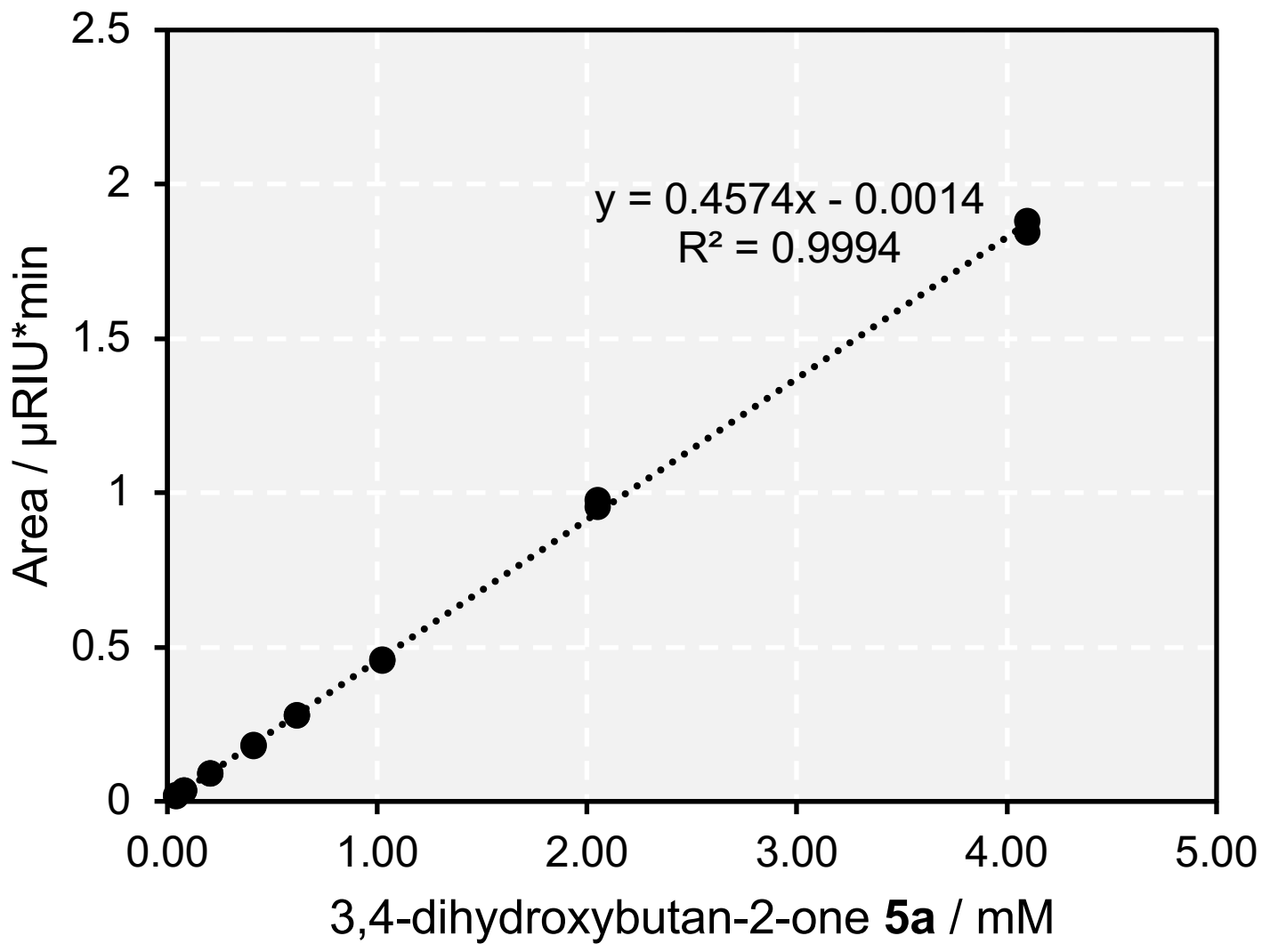


Figure S11. Calibration curve of 3,4-dihydroxybutan-2-one **5a** (HPLC-RI).

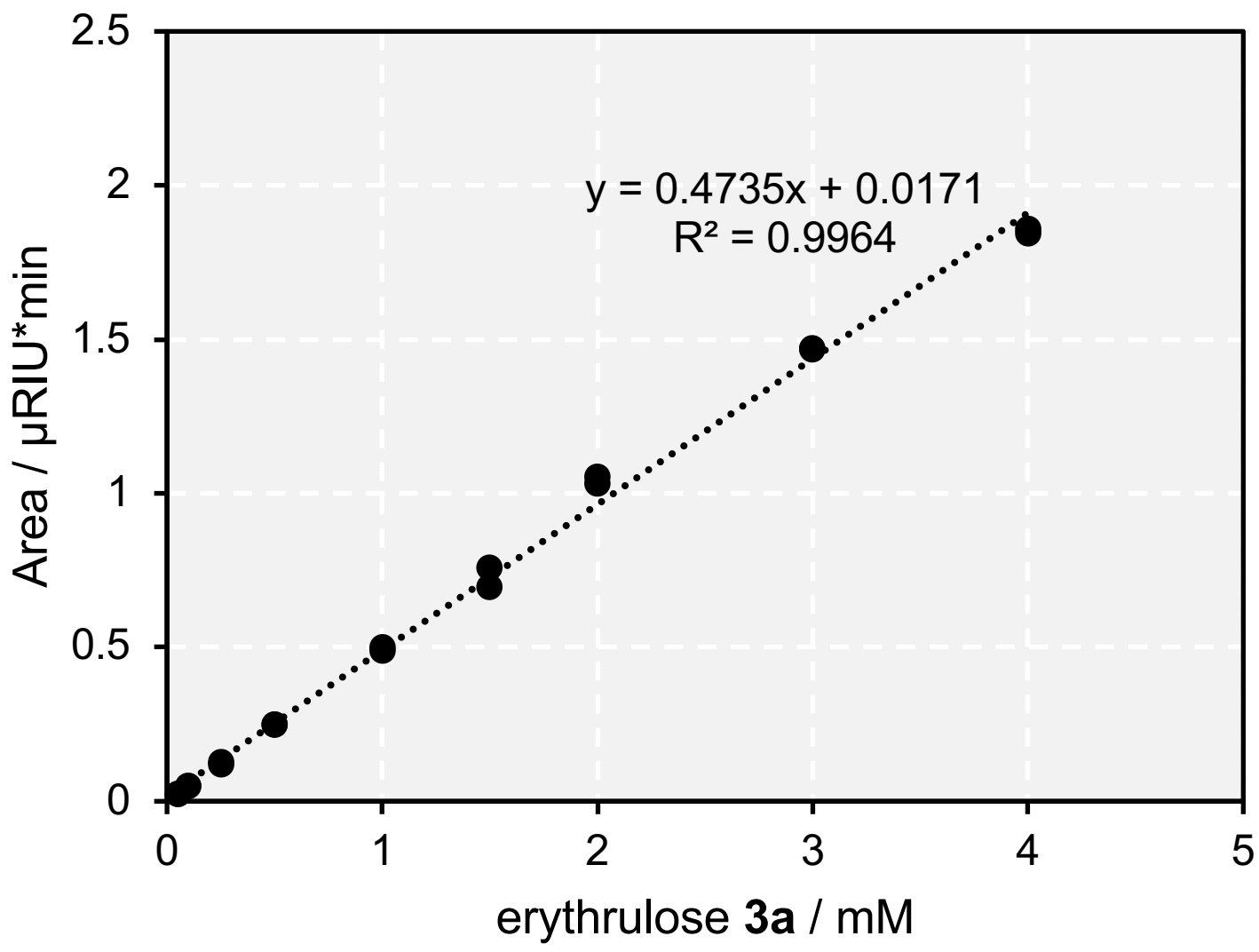


Figure S12. Calibration curve of erythrulose **3a** (HPLC-RI).

## References


1 A. Tonoli, S. Anselmi, J. M. Ward, H. C. Hailes and J. W. E. Jeffries, *ChemBioChem*, 2025, **26**, e202401028.

2 A. Van de Steen, PhD Thesis, University College London, 2023.

3 M. Bawn, F. Subrizi, G. J. Lye, T. D. Sheppard, H. C. Hailes and J. M. Ward, *Data Brief*, 2018, **19**, 1341–1354.

4 M. Bawn, F. Subrizi, G. J. Lye, T. D. Sheppard, H. C. Hailes and J. M. Ward, *Enzyme and Microbial Technology*, 2018, **116**, 16–22.

5 M. E. B. Smith, E. G. Hibbert, A. B. Jones, P. A. Dalby and H. C. Hailes, *Advanced Synthesis & Catalysis*, 2008, **350**, 2631–2638.

6 C. Zhou, T. Saravanan, M. Lorillière, D. Wei, F. Charmantray, L. Hecquet, W. D. Fessner and D. Yi, *ChemBioChem*, 2017, **18**, 455–459.

7 M. E. B. Smith, K. Smithies, T. Senussi, P. A. Dalby and H. C. Hailes, *European Journal of Organic Chemistry*, 2006, **2006**, 1121–1123.

8 A. Cazares, J. L. Galman, L. G. Crago, M. E. Smith, J. Strafford, L. Rios-Solis, G. J. Lye, P. A. Dalby and H. C. Hailes, *Organic & Biomolecular Chemistry*, 2010, **8**, 1301–1309.

9 J. L. Galman, D. Steadman, S. Bacon, P. Morris, M. E. B. Smith, J. M. Ward, P. A. Dalby and H. C. Hailes, *Chemical Communications*, 2010, **46**, 7608–7610.

10 S. A. Kholodar and A. S. Murkin, *Biochemistry*, 2013, **52**, 2302–2308.
